# The Willmore Center of Mass of Initial Data Sets

**DOI:** 10.1007/s00220-022-04349-2

**Published:** 2022-04-08

**Authors:** Michael Eichmair, Thomas Koerber

**Affiliations:** University of Vienna, Faculty of Mathematics, Oskar-Morgenstern-Platz 1, 1090 Vienna, Austria

## Abstract

We refine the Lyapunov–Schmidt analysis from our recent paper (Eichmair and Koerber in Large area-constrained Willmore surfaces in asymptotically Schwarzschild 3-manifolds. arXiv preprint arXiv:2101.12665, 2021) to study the geometric center of mass of the asymptotic foliation by area-constrained Willmore surfaces of initial data for the Einstein field equations. If the scalar curvature of the initial data vanishes at infinity, we show that this geometric center of mass agrees with the Hamiltonian center of mass. By contrast, we show that the positioning of large area-constrained Willmore surfaces is sensitive to the distribution of the energy density. In particular, the geometric center of mass may differ from the Hamiltonian center of mass if the scalar curvature does not satisfy additional asymptotic symmetry assumptions.

## Introduction

Let (*M*, *g*) be an asymptotically flat Riemannian 3-manifold. Such Riemannian manifolds are used to model initial data of isolated gravitational systems for the Einstein field equations. The scalar curvature of (*M*, *g*) provides a lower bound for the local energy density of the initial data set. The geometry of (*M*, *g*) encodes global invariants of the evolving gravitating system.

Recall that the mass $m\in {\mathbb {R}}$ of such a manifold (*M*, *g*), proposed by Arnowitt, Deser, and Misner [1], can be computed as a limit of flux integrals. More precisely,

1 $$\begin{aligned} m=\lim _{\lambda \rightarrow \infty }\sum _{i,\,j=1}^3\,\frac{1}{16\,\pi }\, \lambda ^{-1}\int _{S_\lambda (0)}x^j\,\left[ (\partial _ig)(e_i,e_j)-(\partial _jg)(e_i,e_i)\right] \,\mathrm {d}{\bar{\mu }} \end{aligned}$$

where the integrals are computed in an asymptotically flat chart of (*M*, *g*). Bartnik [2] has shown that the limit in the definition of (1) exists and does not depend on the particular choice of chart. If (*M*, *g*) has non-negative scalar curvature and is not isometric to ${\mathbb {R}}^3$, Schoen and Yau [28] and Witten [29] have shown that $m>0$. The Hamiltonian center of mass associated with (*M*, *g*), proposed by Regge and Teitelboim [27] and by Beig and Ó Murchadha [4], is then given by $C=(C^1,C^2,C^3)$ where

2 $$\begin{aligned} \begin{aligned} C^\ell =\,&\lim _{\lambda \rightarrow \infty }\frac{1}{16\,\pi \,m}\,\lambda ^{-1}\, \int _{S_\lambda (0)}\bigg (\,\sum _{i,\,j=1}^3x^\ell \,x^j\,\big [(\partial _ig) (e_i,e_j)-(\partial _jg)(e_i,e_i)\big ] \\ {}&\qquad -\sum _{i=1}^3\big [x^i\,g(e_i,e_\ell ) -x^\ell \,g(e_i,e_i)\big ]\bigg )\,\mathrm {d}{\bar{\mu }} \end{aligned} \end{aligned}$$

provided the limit exists for each $\ell =1,\,2,\,3$. These limits are known to exist if if *g* satisfies certain asymptotic symmetry conditions; see Theorem 24 below. By contrast, as observed in [27], the limit in (2) may not exist if *g* does not satisfy such additional assumptions. Explicit examples of asymptotically flat initial data with divergent center of mass have been constructed by Beig and Ó Murchadha [4], by Huang [18], and by Cederbaum and Nerz [7].

Let $\Sigma \subset M$ be a closed, two-sided surface with designated outward normal $\nu$ and corresponding mean curvature *H*. The Hawking mass of $\Sigma$ is the quantity

3 $$\begin{aligned} m_H(\Sigma )=\sqrt{\frac{|\Sigma |}{16\,\pi }}\bigg (1-\frac{1}{16\,\pi }\int _{\Sigma } H^2\,\mathrm {d}\mu \bigg ). \end{aligned}$$

To qualify as a quasi-local mass in the sense of [3, p. 235], one would expect that the Hawking mass both detects the local energy distribution and recovers global physical quantities such as the mass (1) and the center of mass (2) of the initial data set as asymptotic limits; see also [25, p. 636]. In [19], Huisken and Ilmanen have proved the Riemannian Penrose inequality by comparing the Hawking mass of an outermost minimal surface to that of a large coordinate sphere in the end of (*M*, *g*) using inverse mean curvature flow. By contrast, the Hawking mass of a closed surface $\Sigma \subset {\mathbb {R}}^3$ is negative unless $\Sigma$ is a round sphere. As a measure of the gravitational field, the quantity $m_H(\Sigma )$ is therefore not appropriate unless $\Sigma$ is in some way special.

As discussed in e.g. [12], there are two classes of surfaces that are particularly well-adapted to the Hawking mass:

$$\circ $$
stable constant mean curvature spheres
$$\circ $$
area-constrained Willmore spheres

In [10], Christodoulou and Yau have observed that stable constant mean curvature spheres have non-negative Hawking mass if (*M*, *g*) has non-negative scalar curvature. Meanwhile, area-constrained Willmore surfaces are by definition critical points of the Hawking mass with respect to an area constraint and thus potential maximizers of the Hawking mass among domains with a prescribed amount of perimeter. These surfaces satisfy the constrained Willmore equation

4 $$\begin{aligned} \Delta H+(|\mathring{h}|^2+{\text {Ric}}(\nu ,\nu )+\kappa )\,H=0. \end{aligned}$$

Here, $\Delta$ is the non-positive Laplace-Beltrami operator with respect to the induced metric on $\Sigma$, $\mathring{h}$ the traceless part of the second fundamental form *h*, ${\text {Ric}}$ the Ricci curvature of (*M*, *g*), and $\kappa \in {\mathbb {R}}$ a Lagrange multiplier. Note that area-constrained Willmore surfaces are also area-constrained critical points of the Willmore energy

$$\begin{aligned} \frac{1}{4}\int _{\Sigma } H^2\,\mathrm {d}\mu . \end{aligned}$$

Lamm, Metzger, and Schulze have studied foliations of asymptotically flat Riemannian 3-manifolds by area-constrained Willmore surfaces and investigated the monotonicity properties of the Hawking mass along this foliation; see [22, Theorem 1, Theorem 2, and Theorem 4] and also the subsequent work [21] of the second-named author. Results analogous to those in [22] but in a space-time setting have been obtained by Friedrich [15].

There have been many recent developments on large stable constant mean curvature spheres in asymptotically flat manifolds. In particular, it is known that the end of every asymptotically flat 3-manifold (*M*, *g*) with non-negative scalar curvature is foliated by large isoperimetric surfaces. This foliation detects the Hamiltonian center of mass (2) of (*M*, *g*) in a natural way. We provide a brief survey of these results in “Appendix B”.

By comparison, much less is known about area-constrained Willmore surfaces. To describe recent developments, given an integer $k\ge 2$, we say that (*M*, *g*) is $C^k$-asymptotic to Schwarzschild with mass $m>0$ if there is a non-empty compact subset of *M* whose complement is diffeomorphic to $\{x\in {\mathbb {R}}^3:|x|>1\}$ and such that, in this so-called asymptotically flat chart of the end of (*M*, *g*), there holds, for every multi-index *J* with $|J|\le k$ and as $x\rightarrow \infty$,

5 $$\begin{aligned} g=\bigg (1+\frac{m}{2\,|x|}\bigg )^4{\bar{g}}+\sigma \qquad \text {with}\qquad \partial _J\sigma =O(|x|^{-2-|J|}). \end{aligned}$$

Here, ${\bar{g}}$ is the Euclidean metric on ${\mathbb {R}}^3$. Note that (*M*, *g*) is modeled upon the initial data of a Schwarzschild black hole. Given such a manifold (*M*, *g*), we fix an asymptotically flat chart and use $B_r$, where $r>1$, to denote the open, bounded domain in (*M*, *g*) whose boundary corresponds to $S_r(0)$ with respect to this chart.

In our recent paper [12], we have established the following existence and uniqueness result. For its statement, recall that the area radius $\lambda (\Sigma )>0$ of a closed surface $\Sigma \subset M$ is defined by

$$\begin{aligned} 4\,\pi \, \lambda (\Sigma )^2=|\Sigma | \end{aligned}$$

while the inner radius $\rho (\Sigma )$ of such a surface is defined by

$$\begin{aligned} \rho (\Sigma )=\sup \{r>1: B_{r}\cap \Sigma =\emptyset \}. \end{aligned}$$

### Theorem 1

[12, Theorems 5 and 8]. Suppose that (*M*, *g*) is $C^4$-asymptotic to Schwarzschild with mass $m>0$ and that its scalar curvature *R* satisfies, as $x\rightarrow \infty$,

$$\begin{aligned} \sum _{i=1}^3x^i\,\partial _i(|x|^2\,R)\le o(|x|^{-2})\qquad \text {and}\qquad R(x)-R(-x)=o(|x|^{-4}). \end{aligned}$$

Then there exist numbers $\kappa _0>0$ and $\epsilon _0>0$ and a family of stable area-constrained Willmore spheres

6 $$\begin{aligned} \{\Sigma (\kappa ):\kappa \in (0,\kappa _0)\} \end{aligned}$$

that foliate the complement of a compact subset of *M* and such that each sphere $\Sigma (\kappa )$ satisfies (4) with parameter $\kappa$. Moreover, given $\delta >0$, there exists a number $\lambda _0>1$ such that every area-constrained Willmore sphere $\Sigma \subset M$ with

$$\begin{aligned} \delta \,\lambda (\Sigma )<\rho (\Sigma ),\qquad \delta \,\rho (\Sigma )<\lambda (\Sigma ),\qquad |\Sigma |>4\,\pi \,\lambda _0^2,\qquad \text {and}\qquad \int _{\Sigma }|\mathring{h}|^2\,\mathrm {d}\mu <\epsilon _0 \end{aligned}$$

satisfies $\Sigma =\Sigma (\kappa )$ for some $\kappa \in (0,\kappa _0)$.

The canonical foliation by area-constrained Willmore surfaces given in Theorem 1 gives rise to a new notion of geometric center of mass,

$$\begin{aligned} C_{ACW}=(C_{ACW}^1,C_{ACW}^2,C_{ACW}^3), \end{aligned}$$

where

7 $$\begin{aligned} C_{ACW}^\ell =\lim _{\kappa \rightarrow 0} |\Sigma (\kappa )|^{-1}\int _{\Sigma (\kappa )} x^\ell \,\mathrm {d}\mu \end{aligned}$$

provided this limit exists for each $\ell =1,\,2,\,3$.

### Outline of the results

Our first main result in this paper shows that the geometric center of mass of the foliation (6) exists and agrees with the Hamiltonian center of mass (2) of (*M*, *g*) if the scalar curvature is sufficiently symmetric with respect to the Hamiltonian center of mass.

#### Theorem 2

Let (*M*, *g*) be $C^4$-asymptotic to Schwarzschild with mass $m>0$ and Hamiltonian center of mass $C=(C^1,\,C^2,\,C^3)$ and suppose that the scalar curvature satisfies, as $x\rightarrow \infty$,

8 $$\begin{aligned} \sum _{i=1}^3{\tilde{x}}^i\,\partial _i(|{\tilde{x}}|^2\, R({\tilde{x}}))&\le o(|x|^{-3}), \end{aligned}$$

9 $$\begin{aligned} R({\tilde{x}})-R(-{\tilde{x}})&=o(|x|^{-5}), \end{aligned}$$

where ${\tilde{x}}=x-C$. Then $C_{ACW}$ exists and $C=C_{ACW}.$

In particular, if $R=0$ outside a compact set, then $C_{ACW}$ exists and equals the Hamiltonian center of mass *C*.

#### Remark 3

According to Theorem 24 and Remark 25, if

$$\begin{aligned} R(x)-R(-x)=O(|x|^{-5}), \end{aligned}$$

then *C* exists.

#### Remark 4

The assumptions (8) and (9) of Theorem 2 hold if, for instance,

$$\begin{aligned} R=o(|x|^{-4})\qquad \text {and} \qquad R(x)-R(-x)=o(|x|^{-5}); \end{aligned}$$

see the argument leading to (31).

The following result shows that the assumptions (8) and (9) in Theorem 2 cannot be relaxed in any substantial way.

#### Theorem 5

There exists a Riemannian 3-manifold (*M*, *g*) that is $C^k$-asymptotic to Schwarzschild with mass $m=2$ for every $k\ge 2$ and satisfies, for every multi-index *J* and as $x\rightarrow \infty$,

$$\begin{aligned} \partial _J\sigma =O(|x|^{-3-|J|}) \end{aligned}$$

such that the Hamiltonian center of mass exists while the limit in (7) does not exist.

Theorem 5 and its proof show that, in general, the positioning of the foliation (6) is not governed by the Hamiltonian center of mass of (*M*, *g*) but instead fine-tuned to the asymptotic distribution of scalar curvature; see Remark 22. By contrast, the positioning of large stable constant mean curvature spheres is not sensitive to the distribution of scalar curvature; see Remark 29. This suggests that large area-constrained Willmore spheres are better suited to detect the local energy distribution of an initial data set than large stable constant mean curvature spheres.

In the second part of this paper, we lay the foundation to investigate the interplay between the positioning of area-constrained Willmore surfaces and the asymptotic distribution of the scalar curvature more thoroughly by extending Theorem 1 to manifolds (*M*, *g*) that are asymptotic to Schwarzschild but whose scalar curvature does not exhibit any asymptotic symmetries beyond those implied by (5).

#### Theorem 6

Let (*M*, *g*) be $C^4$-asymptotic to Schwarzschild with mass $m>0$ and scalar curvature *R* satisfying, as $x\rightarrow \infty$,

10 $$\begin{aligned} R\ge -o(|x|^{-4}). \end{aligned}$$

There exist a number $\kappa _0>0$ and a family $\{\Sigma (\kappa ):\kappa \in (0,\kappa _0)\}$ of area-constrained Willmore spheres $\Sigma (\kappa )$ such that $\Sigma (\kappa )$ satisfies (4) with parameter $\kappa$ and

$$\begin{aligned}&\lim _{\kappa \rightarrow 0} \rho (\Sigma (\kappa ))=\infty , \qquad \limsup _{\kappa \rightarrow 0} \rho (\Sigma (\kappa ))^{-1}\,\lambda (\Sigma (\kappa ))<\infty , \qquad \text{ and }\\&\lim _{\kappa \rightarrow 0} \int _{\Sigma (\kappa )} |\mathring{h}|^2\,\mathrm {d}\mu =0. \end{aligned}$$

Moreover, if the scalar curvature satisfies, as $x\rightarrow \infty$,

11 $$\begin{aligned} \sum _{i=1}^3 x^i\,\partial _i(|x|^2\,R)&\le o(|x|^{-2}), \end{aligned}$$

then there exists a number $\epsilon _0>0$ with the following property. Given $\delta >0$, there exists a number $\lambda _0>1$ such that every area-constrained Willmore sphere $\Sigma \subset M$ with

12 $$\begin{aligned} \begin{aligned}&\delta \,\lambda (\Sigma )<\rho (\Sigma ), \qquad \delta \,\rho (\Sigma )<\lambda (\Sigma ), \qquad |\Sigma |>4\,\pi \,\lambda _0^2, \qquad \text {and} \\&\int _{\Sigma }|\mathring{h}|^2\,\text {d}\mu <\epsilon _0 \end{aligned} \end{aligned}$$

satisfies $\Sigma =\Sigma (\kappa )$ for some $\kappa \in (0,\kappa _0)$.

#### Remark 7

The assumption $\delta \,\rho (\Sigma )<\lambda (\Sigma )$ in (12) can be dropped if one replaces (11) by the stronger condition

$$\begin{aligned} \sum _{i=1}^3x^i\,\partial _i(|x|^2\,R)\le 0; \end{aligned}$$

see [12, Theorem 11].

#### Remark 8

Note that (10) follows from (11).

#### Remark 9

Comparing Theorem 1 and Theorem 6, it is tempting to conjecture that the asymptotic family $\{\Sigma (\kappa ):\kappa \in (0,\kappa _0)\}$ from Theorem 6 forms a foliation. A closer analysis shows that the foliation property of this family depends on the asymptotic behavior of the scalar curvature in a delicate way. We plan to investigate this dependence in a future paper.

**Fig. 1:**
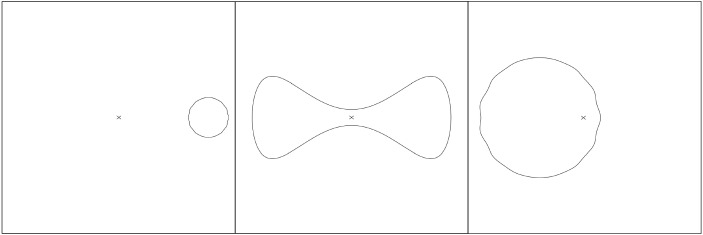
An illustration of the assumptions (12) in the uniqueness statement of Theorem 6. The cross indicates the origin in the asymptotically flat chart. The surface on the left violates the assumption $\rho (\Sigma )<4\,\lambda (\Sigma )$. The surface on the right violates the assumption $\lambda (\Sigma )<4\,\rho (\Sigma )$. The surface in the middle violates the small energy assumption

The assumptions on the scalar curvature in Theorem 6 cannot be relaxed. On the one hand, the uniqueness statement fails if assumption (11) is dropped; see [12, Theorem 13]. On the other hand, we show in the following that the existence of large area-constrained Willmore spheres with comparable area radius and inner radius as well as small energy cannot be guaranteed if the scalar curvature is allowed to change signs.

#### Theorem 10

There exists a Riemannian 3-manifold (*M*, *g*) that is $C^k$-asymptotic to Schwarzschild with mass $m=2$ for every $k\ge 2$ with the following property. There exists no family $\{\Sigma (\kappa ):\kappa \in (0,\kappa _0)\}$ of area-constrained Willmore spheres $\Sigma (\kappa )$ that enclose $B_2$ and satisfy (4) with parameter $\kappa$ such that

$$\begin{aligned}&\lim _{\kappa \rightarrow 0} \rho (\Sigma (\kappa ))=\infty ,\qquad \limsup _{\kappa \rightarrow 0} \rho (\Sigma (\kappa ))^{-1}\,\lambda (\Sigma (\kappa ))<\infty ,\qquad \text {and}\qquad \\&\lim _{\kappa \rightarrow 0} \int _{\Sigma (\kappa )}|\mathring{h}|^2\,\mathrm {d}\mu =0. \end{aligned}$$

Theorem 6 and Remark 7 do not preclude the possibility of a sequence $\{\Sigma _i\}_{i=1}^\infty$ of large area-constrained Willmore spheres $\Sigma _i\subset M$ with

$$\begin{aligned} \lim _{i\rightarrow \infty } \int _{\Sigma _i} |\mathring{h}|^2\,\mathrm {d}\mu =0 \end{aligned}$$

that are slowly divergent in the sense that

$$\begin{aligned} \lim _{i\rightarrow \infty } \rho (\Sigma _i)=\infty \qquad \text {but}\qquad \lim _{i\rightarrow \infty } \rho (\Sigma _i)^{-1}\,\lambda (\Sigma _i)=\infty . \end{aligned}$$

As we discuss in [12], it is a challenging analytical problem to rule out such sequences. Theorem 11 below confirms that the existence of such a sequence hinges on the asymptotic behavior of the scalar curvature, too. It should be compared with the uniqueness result obtained by Qing and Tian [26] for large stable constant mean curvature spheres.

#### Theorem 11

There exists a Riemannian 3-manifold (*M*, *g*) that is $C^k$-asymptotic to Schwarzschild with mass $m=2$ for every $k\ge 2$ such that the following holds. There exists a sequence $\{\Sigma _i\}_{i=1}^\infty$ of area-constrained Willmore spheres $\Sigma _i\subset M$ enclosing $B_2$ such that

$$\begin{aligned} \lim _{i\rightarrow \infty } \rho (\Sigma _i)=\infty , \end{aligned}$$

$\lambda (\Sigma _i)^{-1}\,\Sigma _i$ converges smoothly to a round sphere, but

$$\begin{aligned} \lim _{i\rightarrow \infty } \rho (\Sigma _i)^{-1}\,\lambda _i(\Sigma _i)=\infty \end{aligned}$$

and $m_H(\Sigma _i)>2$ for every *i*.

### Outline of the paper

In order to prove Theorems 2, 5,  6, 10, and 11, we refine the Lyapunov–Schmidt analysis developed in our recent paper [12]. The method of Lyapunov–Schmidt analysis has previously been used by Brendle and the first-named author [5] and by Chodosh and the first-named author [8] to study large stable constant mean curvature speres in Riemannian 3-manifolds asymptotic to Schwarzschild. Contrary to the area-functional under a volume constraint, the Willmore energy under an area constraint is translation invariant up to lower-order terms in exact Schwarzschild; see Lemma 32. New difficulties owing to the competing contributions of the Schwarzschild background respectively the lower-order perturbation of the metric $\sigma$ arise when studying the center of mass of large area-constrained Willmore spheres.

By scaling, we may assume throughout that $m=2$. Geometric computations are performed in the asymptotically flat chart (5). We use a bar to indicate that a geometric quantity has been computed with respect to the Euclidean background metric ${\bar{g}}$. When the Schwarzschild metric

$$\begin{aligned} g_S=(1+|x|^{-1})^4\,{\bar{g}} \end{aligned}$$

with mass $m=2$ has been used in the computation, we use the subscript *S*.

Let $\delta \in (0,1/2)$. In [12], we have used the implicit function theorem to construct surfaces $\Sigma _{\xi ,\lambda }$ as perturbations of $S_\lambda (\lambda \,\xi )$ where $\xi \in {\mathbb {R}}^3$ with $|\xi |<1-\delta$ and $\lambda >1$ is large such that $|\Sigma _{\xi ,\lambda }|=4\,\pi \,\lambda ^2$ and $\Sigma _{\xi ,\lambda }$ is an area-constrained Willmore surface if and only if $\xi$ is a critical point of the function $G_\lambda$ defined by

$$\begin{aligned} G_\lambda (\xi )=\lambda ^2\bigg (\int _{\Sigma _{\xi ,\lambda }} H^2\,\mathrm {d}\mu -16\,\pi +64\,\pi \,\lambda ^{-1}\bigg ). \end{aligned}$$

We have also proven that

$$\begin{aligned} G_\lambda (\xi )=G_1(\xi ) +G_{2,\lambda }(\xi )+ G_{3,\lambda }(\xi ) \end{aligned}$$

where $G_1$ is a rotationally symmetric and strictly convex function independent of $\lambda$,

$$\begin{aligned} G_{2,\lambda }(\xi )= 2\,\lambda \int _{{\mathbb {R}}^3\setminus {B_{\lambda }(\lambda \,\xi )}} R\,\mathrm {d}\bar{v}, \end{aligned}$$

and $G_{3,\lambda }=O(\lambda ^{-1})$ as $\lambda \rightarrow \infty$. Here, *R* is the scalar curvature of (*M*, *g*). We refer to “Appendix C” for more details on this construction.

Under the assumptions of Theorem 1, we have shown in [12] that the function $G_\lambda$ has a unique critical point $\xi (\lambda )\in {\mathbb {R}}^3$ with $\xi (\lambda )=o(1)$ as $\lambda \rightarrow \infty$. The sphere $\Sigma _{\xi (\lambda ),\lambda }$ corresponds to a leaf $\Sigma (\kappa )$ of the foliation (6) for suitable $\kappa =\kappa (\lambda )$. Moreover, we observe that

$$\begin{aligned} \lambda \,\xi (\lambda )=|\Sigma (\kappa (\lambda ))|^{-1}\int _{\Sigma (\kappa (\lambda ))} x^\ell \,\mathrm {d}\mu +O(\lambda ^{-1}). \end{aligned}$$

On the one hand, we compute here that $\lambda \,({\bar{D}}G_{3,\lambda })({\xi (\lambda )})$ is essentially proportional to the Hamiltonian center of mass *C* provided $\lambda >1$ is sufficiently large. On the other hand, we prove that $\lambda \,({\bar{D}} G_{2,\lambda })({\xi (\lambda )})$ is small if the scalar curvature satisfies the assumptions of Theorem 2. Since $\xi (\lambda )$ is a critical point of $G_\lambda$, this proves Theorem 2. By contrast, we show by explicit example that $\lambda \,({\bar{D}} G_{2,\lambda })({\xi (\lambda )})$ need not converge as $\lambda \rightarrow \infty$ if the assumptions on the scalar curvature are relaxed only slightly. This proves Theorem 5.

To prove Theorem 6, we use a geometric argument to show that the function $G_{2,\lambda }$ is convex if the scalar curvature satisfies the growth condition (11). In particular, the function $G_\lambda$ has a critical point $\xi (\lambda )$ that is unique among all $\xi \in {\mathbb {R}}^3$ with $|\xi |<1-\delta$ provided $\lambda >1$ is sufficiently large. By contrast, we construct a metric whose scalar curvature changes signs such that for some sequence $\{\lambda _i\}_{i=1}^\infty$ with $\lim _{i\rightarrow \infty }\lambda _i=\infty$ and every $\delta >0$ there are infinitely many *i* for which the function $G_{\lambda _i}$ has no critical points $\xi \in {\mathbb {R}}^3$ with $|\xi |<1-\delta$. Likewise, we construct a metric such that for every $\delta >0$ there are infinitely many *i* for which $G_{\lambda _i}$ has a critical point $\xi _i \in {\mathbb {R}}^3$ with $1-\delta<|\xi _i|<1$. This proves Theorems 10 and 11.

## Proof of Theorems 2 and 5

Throughout this section, we assume that (*M*, *g*) is $C^4$-asymptotic to Schwarzschild with mass $m=2$ and scalar curvature *R* satisfying

13 $$\begin{aligned} \sum _{i=1}^3x^i\,\partial _i(| x|^2\, R)&\le O(|x|^{-3}), \nonumber \\ R(x)-R(-x)&=O(|x|^{-5}) . \end{aligned}$$

To prove Theorem 2, we expand upon the Lyapunov–Schmidt analysis developed in our recent work [12]. The required concepts and estimates are summarized in “Appendix C”.

Let $\delta =1/4$, $\lambda _0>1$ be the constant from Proposition 31, and $\xi \in {\mathbb {R}}^3$ with $|\xi |<3/4$. Recall the definitions (69) of the function $G_\lambda$, (68) of the surface $\Sigma _{\xi ,\lambda }$, and (65) of the sphere $S_{\xi ,\lambda }$. Let $\xi (\lambda )$ be the unique critical point of $G_\lambda$ with $|\xi (\lambda )|<3/4$ whose existence is asserted in Proposition 35.

Recall the Lagrange parameter $\kappa$ defined in (67). It follows from Propositions 35, 31, and Remark 36 that $\Sigma _{\xi (\lambda ),\lambda }$ is the area-constrained Willmore sphere $\Sigma (\kappa )$ from (6) with $\kappa =\kappa (\Sigma (\lambda ))$.

### Lemma 12

There holds, as $\lambda \rightarrow \infty$,

$$\begin{aligned} \xi (\lambda )=O(\lambda ^{-1}). \end{aligned}$$

### Proof

Using Lemma 32, Lemma 34, and that $({\bar{D}}G_\lambda )({\xi (\lambda )})=0$, we find

$$\begin{aligned} 0=|\xi (\lambda )|^{-1}\,\sum _{i=1}^3\xi (\lambda )^i(\partial _i G_\lambda )({\xi (\lambda )})\ge |\xi (\lambda )|^{-1}\,\sum _{i=1}^3\xi (\lambda )^i\,(\partial _i G_{1})({\xi (\lambda )})-O(\lambda ^{-1}). \end{aligned}$$

Using (73), we obtain

$$\begin{aligned} |\xi (\lambda )|^{-1}\,\sum _{i=1}^3\xi (\lambda )^i\,(\partial _i G_{1})({\xi (\lambda )})\ge 256\,\pi \,|\xi (\lambda )|. \end{aligned}$$

The assertion of the lemma follows from combining these estimates. $\square$

Recall from Proposition 31 that $\Sigma _{\xi ,\lambda }=\Sigma _{\xi ,\lambda }(u_{\xi ,\lambda })$, i.e. $\Sigma _{\xi ,\lambda }$ is the radial graph (66) of the function $u_{\xi ,\lambda }$ over $S_{\xi ,\lambda }$. We define

$$\begin{aligned} {\tilde{u}}_{\xi ,\lambda }=u_{\xi ,\lambda }+2 \end{aligned}$$

so that

$$\begin{aligned} \Sigma _{\xi ,\lambda }=\Sigma _{{\tilde{\xi }},{\tilde{\lambda }}}({\tilde{u}}_{\xi ,\lambda }) \end{aligned}$$

with

14 $$\begin{aligned} {\tilde{\lambda }} = \lambda -2\qquad \text {and}\qquad {\tilde{\xi }}=(\lambda -2)^{-1}\,\lambda \,\xi . \end{aligned}$$

Note that $$\lambda \,\xi ={\tilde{\lambda }}\,{\tilde{\xi }}$$.

Recall the vector field $Z_{\xi ,\lambda }$ defined in (78). We abbreviate $u_{\xi ,\lambda },\,{\tilde{u}}_{\xi ,\lambda },\, Z_{\xi ,\lambda },$ and $Z_{{\tilde{\xi }},{\tilde{\lambda }}}$ by $u,\,{\tilde{u}},\,Z$, and ${\tilde{Z}}$, respectively. Moreover, we let $\Lambda _0(S_{{\tilde{\xi }},{\tilde{\lambda }}})\subset C^\infty (S_{{\tilde{\xi }},{\tilde{\lambda }}})$ be the space of constant functions and $\Lambda _0^\perp (S_{{\tilde{\xi }},{\tilde{\lambda }}})$ be its orthogonal complement. We abbreviate $\Lambda _0(S_{{\tilde{\xi }},{\tilde{\lambda }}})$ by $\Lambda _0$ and $\Lambda _0^\perp (S_{{\tilde{\xi }},{\tilde{\lambda }}})$ by $\Lambda _0^\perp$.

### Lemma 13

There exists $\delta \in (0,1/4)$ such that

15 $$\begin{aligned} {\tilde{u}}=O(|\xi |^2)+O(\lambda ^{-1}) \end{aligned}$$

and, uniformly for every $\xi \in {\mathbb {R}}^3$ with $|\xi |<\delta$ as $\lambda \rightarrow \infty$,

16 $$\begin{aligned} {\text {proj}}_{\Lambda _0}{\tilde{u}}=-\lambda ^{-1}-\frac{1}{16\,\pi }\, \lambda ^{-1}\int _{S_{\xi ,\lambda }}[\bar{{\text {tr}}}\,\sigma -\sigma ({\bar{\nu }},{\bar{\nu }})]\,\mathrm {d}{\bar{\mu }}+O(\lambda ^{-2}) +O(\lambda ^{-1}\,|\xi |^2). \end{aligned}$$

These expansions may be differentiated once with respect to $\xi$.

### Proof

(15) follows directly from (70). Using the identity

$$\begin{aligned} \mathrm {d}\mu =\left[ 1+4\,|x|^{-1}+6\,|x|^{-2}+\frac{1}{2}\,(\bar{{\text {tr}}} \,\sigma -\sigma ({\bar{\nu }},{\bar{\nu }}))+O(|x|^{-3})\right] \mathrm {d}{\bar{\mu }}, \end{aligned}$$

(14), and the fact that (*M*, *g*) is $C^4$-asymptotic to Schwarzschild, we find that

$$\begin{aligned} |S_{{\tilde{\xi }},{\tilde{\lambda }}}|=4\,\pi \,\lambda ^2+8\,\pi +\frac{1}{2} \int _{S_{\xi ,\lambda }}[\bar{{\text {tr}}}\,\sigma - \sigma ({\bar{\nu }},{\bar{\nu }})]\,\mathrm {d}{\bar{\mu }}+O(\lambda ^{-1})+O(|\xi |^{2}) \end{aligned}$$

provided that $\delta >0$ is sufficiently small. Using also that $|\Sigma _{\xi ,\lambda }|=4\,\pi \,\lambda ^2$, that $H(S_{{\tilde{\xi }},{\tilde{\lambda }}})=2\,\lambda ^{-1}+O(\lambda ^{-2})$, and (15), the first variation of area formula therefore yields

$$\begin{aligned} 2\,\lambda ^{-1}\,\int _{S_{{\tilde{\xi }},{\tilde{\lambda }}}}{\tilde{u}}\, \mathrm {d}{\bar{\mu }}=\,&|\Sigma _{\xi ,\lambda }|-|S_{{\tilde{\xi }}, {\tilde{\lambda }}}|+O(\lambda ^{-1})+O(|\xi |^{2})\\ =\,&-8\,\pi - \frac{1}{2} \int _{S_{\xi ,\lambda }}[\bar{{\text {tr}}}\,\sigma -\sigma ({\bar{\nu }}, {\bar{\nu }})]\,\mathrm {d}{\bar{\mu }}+O(\lambda ^{-1})+O(|\xi |^{2}). \end{aligned}$$

This implies (16). $$\square $$

We proceed to compute a precise estimate for the Willmore energy of $\Sigma _{\xi ,\lambda }$.

### Lemma 14

There exists $\delta \in (0,1/4)$ such that, uniformly for every $\xi \in {\mathbb {R}}^3$ with $|\xi |<\delta$ as $\lambda \rightarrow \infty$,

$$\begin{aligned} \int _{\Sigma _{\xi ,\lambda }}H^2\,\mathrm {d}\mu =&\,\int _{S_{{\tilde{\xi }}, {\tilde{\lambda }}}} H^2\,\mathrm {d}\mu -64\,\pi \,\lambda ^{-3}-4\,\lambda ^{-3}\,\int _{S_{\xi , \lambda }}[\bar{{\text{ tr }}}\,\sigma -\sigma ({\bar{\nu }},{\bar{\nu }})]\, \mathrm {d}{\bar{\mu }}\\ {}&\quad +O(\lambda ^{-3}\,|\xi |^2)+O(\lambda ^{-2}\,|\xi |^4\,)+O(\lambda ^{-4}). \end{aligned}$$

This expansion may be differentiated once with respect to $\xi$.

### Proof

According to Lemma 40, we have

$$\begin{aligned}&{\text {proj}}_{\Lambda _0}W(S_{{\tilde{\xi }},{\tilde{\lambda }}})= -8\,\lambda ^{-4}+O(\lambda ^{-5})\qquad \text {and}\\&{\text {proj}}_{\Lambda _0^\perp }W(S_{{\tilde{\xi }},{\tilde{\lambda }}}) =O(\lambda ^{-4}\,|\xi |^2)+O(\lambda ^{-5}). \end{aligned}$$

The assertion follows from this, Lemmas 13 and 42. $\square$

### Remark 15

Let $\delta \in (0,1/4)$ and suppose that

$$\begin{aligned} {\mathcal {E}}:\{\xi \in {\mathbb {R}}^3:|\xi |<\delta \}\times \{\lambda \in {\mathbb {R}}: \lambda >1\}\rightarrow {\mathbb {R}} \end{aligned}$$

satisfies, as $$\lambda \rightarrow \infty $$,

$$\begin{aligned} {\mathcal {E}}=O(\lambda ^{-3}\,|\xi |^2)+O(\lambda ^{-2}\,|\xi |^4\,)+O(\lambda ^{-4}) \end{aligned}$$

and

$$\begin{aligned} {\bar{D}}{\mathcal {E}}=O(\lambda ^{-3}\,|\xi |)+O(\lambda ^{-2}\,|\xi |^3\,) +O(\lambda ^{-4}) \end{aligned}$$

where differentiation is with respect to $\xi$. Using Lemma 12, we find that, as $\lambda \rightarrow \infty$,

$$\begin{aligned} ({\bar{D}}{\mathcal {E}})({\xi (\lambda )})=O(\lambda ^{-4}). \end{aligned}$$

### Lemma 16

There exists $\delta \in (0,1/4)$ such that, uniformly for every $\xi \in {\mathbb {R}}^3$ with $|\xi |<\delta$ as $\lambda \rightarrow \infty$,

$$\begin{aligned} \int _{S_{{\tilde{\xi }},{\tilde{\lambda }}}} H_S^2\,\mathrm {d}\mu _S=16\,\pi -64\,\pi \,\lambda ^{-1}+128\,\pi \,|\xi |^2\, \lambda ^{-2}+O(\lambda ^{-3}\,|\xi |^2)+O(\lambda ^{-4}). \end{aligned}$$

This expansion may be differentiated once with respect to $\xi$.

### Proof

This follows from (14) and a direct computation similar to that in [12, Lemma 42]. $\square$

Recall the conformal Killing operator ${\mathcal {D}}$ defined in (79).

### Lemma 17

There exists $\delta \in (0,1/4)$ such that, uniformly for every $\xi \in {\mathbb {R}}^3$ with $|\xi |<\delta$ as $\lambda \rightarrow \infty$,

$$\begin{aligned}&\int _{S_{{\tilde{\xi }},{\tilde{\lambda }}}} H^2\,\mathrm {d}\mu -\int _{S_{{\tilde{\xi }},{\tilde{\lambda }}}} H_S^2\,\mathrm {d}\mu _S \\ {}&\quad =\,2\,{{\tilde{\lambda }}}^{-1}\int _{{\mathbb {R}}^3\setminus B_{{\tilde{\lambda }}}(\lambda \,\xi )} R\,\mathrm {d}{\bar{v}} \\ {}&\qquad \quad +8\,\lambda ^{-3}\int _{S_{\xi ,\lambda }}\bigg [(\sigma ({\bar{\nu }}, {\bar{\nu }})-6\,{\bar{g}}(\xi ,{\bar{\nu }}))\,\sigma ({\bar{\nu }},{\bar{\nu }}) +3\,\sigma ({\bar{\nu }},\xi )\bigg ]\,\mathrm {d}{\bar{\mu }} \\ {}&\qquad \quad -4\,\lambda ^{-1}\,\int _{{\mathbb {R}}^3\setminus B_\lambda (\lambda \,\xi )}|x|^{-3} \,\sum _{i=1}^3\bigg [2\,({\bar{D}}^2_{e_i,x}\sigma )(e_i,x)-({\bar{D}}^2_{e_i,e_i} \sigma )(x,x)\\ {}&\qquad \qquad -({\bar{D}}^2_{x,x}\sigma )(e_i,e_i) +\sum _{j=1}^3\big [({\bar{D}}^2_{e_i,e_i}\sigma )(e_j,e_j)-({\bar{D}}^2_{e_i,e_j} \sigma )(e_i,e_j)\big ]\bigg ]\,\mathrm {d}{\bar{\mu }} \\ {}&\qquad \quad +4\int _{{\mathbb {R}}^3\setminus B_\lambda (\lambda \,\xi )}|x|^{-3} \,\sum _{i=1}^3\,\bigg [({\bar{D}}^2_{e_i,x}\sigma )(e_i,\xi )+ ({\bar{D}}^2_{e_i,\xi }\sigma )(e_i,x) \\ {}&\qquad \qquad -({\bar{D}}^2_{e_i,e_i}\sigma )(x,\xi )-(\bar{D}^2_{x,\xi }\sigma )(e_i,e_i)\bigg ]\,\mathrm {d}{\bar{\mu }} \\ {}&\qquad \quad -4\int _{{\mathbb {R}}^3\setminus B_\lambda (\lambda \,\xi )} |x|^{-3}\bigg [\lambda ^{-1}{\bar{D}}_x\bar{{\text{ tr }}}\,\sigma -3\,\lambda ^{-1}\,|x|^{-2}\,({\bar{D}}_x\sigma )(x,x)\\ {}&\qquad \qquad -{\bar{D}}_\xi \bar{{\text{ tr }}}\,\sigma +3\,|x|^{-2}\,({\bar{D}}_\xi \sigma )(x,x)\bigg ] \,\mathrm {d}{\bar{\mu }} \\ {}&\qquad \quad +O(\lambda ^{-4})+O(|\xi |^2\,\lambda ^{-3}). \end{aligned}$$

This expansion may be differentiated once with respect to $\xi$.

### Proof

As in the proof of [12, Lemma 42], there holds

17 $$\begin{aligned} \begin{aligned} \int _{S_{{\tilde{\xi }},{\tilde{\lambda }}}} H^2\,\mathrm {d}\mu =&16\,\pi -64\,\pi \,{\tilde{\lambda }}^{-1} +2\,\int _{S_{{\tilde{\xi }},{\tilde{\lambda }}}} |\mathring{h}|^2\,\mathrm {d}\mu +\frac{2}{3}\int _{{\mathbb {R}}^3\setminus B_{{\tilde{\lambda }}} (\lambda \,\xi )}({\text{ div }} {\tilde{Z}})\, R\, \mathrm {d}v \\ {}&\quad +4\int _{S_{{\tilde{\xi }},{\tilde{\lambda }}}} {\text{ Ric }} (\nu -{\tilde{Z}},\nu )\,\mathrm {d}\mu -2\int _{{\mathbb {R}}^3\setminus B_{{\tilde{\lambda }}}(\lambda \,\xi )} g({\text{ Ric }},{\mathcal {D}}{\tilde{Z}})\,\mathrm {d}v. \end{aligned} \end{aligned}$$

Note that the first integral on the right-hand side is conformally invariant. It follows that

18 $$\begin{aligned} \int _{S_{{\tilde{\xi }},{\tilde{\lambda }}}} |\mathring{h}_S|^2\,\mathrm {d}\mu _S=0. \end{aligned}$$

Likewise, using $R_S=0$, we find that

19 $$\begin{aligned} \int _{{\mathbb {R}}^3\setminus B_{{\tilde{\lambda }}} (\lambda \,\xi )} {\text {div}}_S {\tilde{Z}}\,R_S\,\mathrm {d}v_S =0. \end{aligned}$$

Similarly, we have ${\tilde{Z}}=\nu _S$ on $S_{{\tilde{\xi }},{\tilde{\lambda }}}$ and consequently

20 $$\begin{aligned} \int _{S_{{\tilde{\xi }},{\tilde{\lambda }}}} {\text {Ric}}_S(\nu _S-{\tilde{Z}},\nu _S)\,\mathrm {d}\mu _S=0. \end{aligned}$$

According to Lemma 41, there holds

21 $$\begin{aligned} \int _{S_{{\tilde{\xi }},{\tilde{\lambda }}}} |\mathring{h}|^2\,\mathrm {d}\mu =O(\lambda ^{-4}). \end{aligned}$$

We compute that

$$\begin{aligned} {\text {div}}{\tilde{Z}}\!=\!3\,(1+|x|^{-1})^{-2}\,{\tilde{\lambda }}^{-1} \!-4\,{\tilde{\lambda }}^{-1}\,|x|^{-1}+4\,|x|^{-3}\,{\bar{g}}({\tilde{\xi }},x)+O(\lambda ^{-1}\,|x|^{-2})+O(|x|^{-3}). \end{aligned}$$

In conjunction with the estimate

$$\begin{aligned} \mathrm {d}\mu =[1+4\,|x|^{-1} +O(|x|^{-2})]\,\mathrm {d}{\bar{\mu }}, \end{aligned}$$

it follows that

22 $$\begin{aligned} \int _{{\mathbb {R}}^3\setminus B_{{\tilde{\lambda }}} (\lambda \,\xi )} {\text{ div }} {\tilde{Z}}\,R\, \mathrm {d}v=&{} {\tilde{\lambda }}^{-1} \int _{{\mathbb {R}}^3\setminus B_{{\tilde{\lambda }}} (\lambda \,\xi )} \bigg [3+2\,|x|^{-1}+4\,|x|^{-3}\,{\bar{g}}(\xi ,x)\bigg ]\,R\,\mathrm {d}{\bar{v}}\nonumber \\ {}&\quad +O(\lambda ^{-4})+O(|\xi |^2\,\lambda ^{-3}). \end{aligned}$$

Using (14), (75), and (77), we find

23 $$\begin{aligned} \int _{S_{{\tilde{\xi }},{\tilde{\lambda }}}} {\text{ Ric }}(\nu -{\tilde{Z}},\nu )\,\mathrm {d}\mu =\,&\int _{S_{\xi ,\lambda }} {\text{ Ric }}_S(\nu -Z,{\bar{\nu }})\,\mathrm {d}{\bar{\mu }}+O(\lambda ^{-4}) \nonumber \\=\,&2\,\lambda ^{-3}\int _{S_{\xi ,\lambda }}\bigg [\sigma ({\bar{\nu }},{\bar{\nu }}) -6\,{\bar{g}}(\xi ,{\bar{\nu }})\,\sigma ({\bar{\nu }},{\bar{\nu }})+3\, \sigma ({\bar{\nu }},\xi )\bigg ]\,\mathrm {d}{\bar{\mu }}\nonumber \\ {}&\quad +O(\lambda ^{-4})+O(|\xi |^2\,\lambda ^{-3}). \end{aligned}$$

Using (14), we obtain

24 $$\begin{aligned} \begin{aligned}&\int _{{\mathbb {R}}^3\setminus B_{{\tilde{\lambda }}}(\lambda \,\xi )} g({\text {Ric}},{\mathcal {D}}{\tilde{Z}})\,\mathrm {d}\mu -\int _{{\mathbb {R}}^3\setminus B_{{\tilde{\lambda }}}(\lambda \,\xi )} g_S({\text {Ric}}_S,{\mathcal {D}}_S{\tilde{Z}})\,\mathrm {d}\mu _S\\&\quad =\,\int _{{\mathbb {R}}^3\setminus B_{{\tilde{\lambda }}}(\lambda \,\xi )} \bigg [{\bar{g}}({{\text {Ric}}}-{\text {Ric}}_S,{\mathcal {D}}_S{\tilde{Z}}) +{\bar{g}}({{\text {Ric}}_S},{\mathcal {D}}{\tilde{Z}}- {\mathcal {D}}_S{\tilde{Z}})\bigg ]\,\mathrm {d}{\bar{\mu }} +O(\lambda ^{-4}) \\&\quad =\int _{{\mathbb {R}}^3\setminus B_{ \lambda }(\lambda \,\xi )} \bigg [{\bar{g}}({{\text {Ric}}}-{\text {Ric}}_S,{\mathcal {D}}_SZ) +{\bar{g}}({{\text {Ric}}_S},{\mathcal {D}} Z-{\mathcal {D}}_S Z)\bigg ]\, \mathrm {d}{\bar{\mu }}+O(\lambda ^{-4}). \end{aligned} \end{aligned}$$

Using (76), (80), and that $R_S=0$, we compute

25 $$\begin{aligned} \begin{aligned}&\int _{{\mathbb {R}}^3\setminus B_\lambda (\lambda \,\xi )} {\bar{g}}({{\text{ Ric }}-{\text{ Ric }}_S},{\mathcal {D}}_SZ)\, \mathrm {d}{\bar{\mu }}\\ {}&\quad =\,2\,\lambda ^{-1}\int _{{\mathbb {R}}^3\setminus B_\lambda (\lambda \,\xi )}|x|^{-3}\, \sum _{i=1}^3 \bigg [2\,({\bar{D}}^2_{e_i,x}\sigma )(e_i,x)-({\bar{D}}^2_{e_i,e_i}\sigma )(x,x)\\ {}&\qquad \qquad - ({\bar{D}}^2_{x,x}\sigma )(e_i,e_i)\bigg ]\,\mathrm {d}{\bar{\mu }}\\ {}&\qquad \quad \,-2\int _{{\mathbb {R}}^3\setminus B_\lambda (\lambda \,\xi )}|x|^{-3}\,\sum _{i=1}^3 \bigg [({\bar{D}}^2_{e_i,x}\sigma )(e_i,\xi )+({\bar{D}}^2_{e_i,\xi }\sigma )(e_i,x) \\ {}&\qquad \qquad -({\bar{D}}^2_{e_i,e_i}\sigma )(x,\xi )-({\bar{D}}^2_{x,\xi }\sigma ) (e_i,e_i)\bigg ]\,\mathrm {d}{\bar{\mu }} \\ {}&\qquad \quad \,+\frac{4}{3}\int _{{\mathbb {R}}^3\setminus B_{\lambda }(\lambda \,\xi )}\bigg [|x|^{-3}\,{\bar{g}}(x,\xi )- \lambda ^{-1}\,|x|^{-1}\bigg ]\,R\,\mathrm {d}{\bar{\mu }} \\ {}&\qquad \quad \,+O(\lambda ^{-4}). \end{aligned} \end{aligned}$$

For the first line of the right-hand side of (25), we note that

26 $$\begin{aligned} R=\sum _{i,\,j=1}^3\big [({\bar{D}}^2_{e_i,e_j}\sigma )(e_i,e_j) -{\bar{D}}^2_{e_i,e_i}\sigma (e_j,e_j)\big ]+O(|x|^{-5}); \end{aligned}$$

see (76). Finally, using (81) and that $R_S=0$, we obtain

27 $$\begin{aligned} \begin{aligned}&\int _{{\mathbb {R}}^3\setminus B_\lambda (\lambda \,\xi )} {\bar{g}}({{\text{ Ric }}_S},{{\mathcal {D}}Z}-{\mathcal {D}}_SZ)\, \mathrm {d}{\bar{\mu }}\\ {}&\quad =\,2\int _{{\mathbb {R}}^3\setminus B_\lambda (\lambda \,\xi )} |x|^{-3}\bigg [\lambda ^{-1}({\bar{D}}_x\bar{{\text{ tr }}}\,\sigma ) -3\,\lambda ^{-1}\,|x|^{-2}\,({\bar{D}}_x\sigma )(x,x)-({\bar{D}}_\xi \bar{{\text{ tr }}}\,\sigma )\\&\qquad \qquad \quad +3\,|x|^{-2}\,({\bar{D}}_\xi \sigma )(x,x)\bigg ] \,\mathrm {d}{\bar{\mu }} \\ {}&\qquad \quad +O(\lambda ^{-4}). \end{aligned} \end{aligned}$$

Assembling (17–27), the assertion of the lemma follows. $\square$

### Proposition 18

There holds, as $\lambda \rightarrow \infty$,

$$\begin{aligned} 256\,\pi \,\lambda \,\xi (\lambda )=&\,2\,\lambda ^3\,\int _{S_{\lambda } (\lambda \,\xi (\lambda ))}R\,{\bar{\nu }}\,\mathrm {d}{\bar{\mu }}\\ {}&\quad + 8\,\lambda \int _{S_\lambda (0)}\left[ (\bar{D}\,\bar{{\text{ tr }}}\, \sigma )-({\bar{D}}\sigma )({\bar{\nu }},{\bar{\nu }})-2\,\lambda ^{-1}\, \bar{{\text{ tr }}}\,\sigma \,{\bar{\nu }}\right] \,\mathrm {d}{\bar{\mu }}\\ {}&\quad +O(\lambda ^{-1}). \end{aligned}$$

### Proof

Recall that $({\bar{D}} G_\lambda )({\xi (\lambda )})=0.$ In conjunction with Lemmas 12 and 14, this implies

28 $$\begin{aligned} \left( {\bar{D}} \int _{S_{\xi ,{\tilde{\lambda }}}} H^2\,\mathrm {d}\mu \right) ({{\tilde{\xi }}(\lambda )})-4\,\lambda ^{-2}\, \int _{S_{\xi ,\lambda }}\big [{\bar{D}}\,\bar{{\text {tr}}}\, \sigma -({\bar{D}}\sigma )({\bar{\nu }},{\bar{\nu }})\big ]\, \mathrm {d}{\bar{\mu }}+O(\lambda ^{-4})=0. \end{aligned}$$

Lemmas 12 and 16 imply that

29 $$\begin{aligned} \left( {\bar{D}} \int _{S_{\xi ,{\tilde{\lambda }}}} H_S^2\,\mathrm {d}\mu _S\right) ({{\tilde{\xi }}(\lambda )})=256\,\pi \, \lambda ^{-2}\,\xi (\lambda )+O(\lambda ^{-4}). \end{aligned}$$

Using (14), Lemma 12, and the fact that (*M*, *g*) is $C^4$-asymptotic to Schwarzschild, we infer from Lemma 17 that, for every $a\in {\mathbb {R}}^3$ with $|a|=1$, we have

30 $$\begin{aligned} \begin{aligned}&\left( {\bar{D}}_a \int _{S_{ \xi ,{\tilde{\lambda }}}} H^2\,\mathrm {d}\mu \right) ({{\tilde{\xi }}(\lambda )})-\left( {\bar{D}}_a \int _{S_{\xi ,{\tilde{\lambda }}}} H_S^2\,\mathrm {d}\mu _S\right) ({{\tilde{\xi }}(\lambda )}) \\ {}&\quad =\,-2 \,\lambda \,{\tilde{\lambda }}\,\int _{S_{{\tilde{\lambda }}}(\lambda \, \xi (\lambda ))}{\bar{g}}({\bar{\nu }},a)\,R\,\mathrm {d}{\bar{\mu }} \\ {}&\qquad \quad +\,8\,\lambda ^{-3}\int _{S_{\lambda }(0)}\bigg [\lambda \,({\bar{D}}_a\sigma ) ({\bar{\nu }},{\bar{\nu }})-6\,{\bar{g}}(a,{\bar{\nu }})\, \sigma ({\bar{\nu }},{\bar{\nu }})+3\,\sigma ({\bar{\nu }},a)\bigg ]\,\mathrm {d}{\bar{\mu }}\\ {}&\qquad \quad +4\,\lambda ^{-1}\,\int _{S_\lambda (0)}{\bar{g}}(\nu ,a)\,\sum _{i=1}^3 \bigg [2\,({\bar{D}}^2_{e_i,{\bar{\nu }}}\sigma )({\bar{\nu }},e_i) -({\bar{D}}^2_{e_i,e_i}\sigma )({\bar{\nu }},{\bar{\nu }})\\ {}&\qquad \qquad -({\bar{D}}^2_{{\bar{\nu }}, {\bar{\nu }}}\sigma )(e_i,e_i) +\sum _{j=1}^3\big [({\bar{D}}^2_{e_i,e_i}\sigma )(e_j,e_j)-({\bar{D}}^2_{e_i,e_j} \sigma )(e_i,e_j)\big ]\bigg ]\,\mathrm {d}{\bar{\mu }} \\ {}&\qquad \quad +4\,\int _{{\mathbb {R}}^3\setminus B_\lambda (0)}|x|^{-3} \,\sum _{i=1}^3\bigg [({\bar{D}}^2_{e_i,x}\sigma )(e_i,a)+({\bar{D}}^2_{e_i,a} \sigma )(e_i,x)\\ {}&\qquad \qquad -({\bar{D}}^2_{e_i,e_i}\sigma )(x,a)-({\bar{D}}^2_{x,a} \sigma )(e_i,e_i)\bigg ]\,\mathrm {d}{\bar{\mu }} \\ {}&\qquad \quad +4\,\lambda ^{-2}\,\int _{S_\lambda (0)} {\bar{g}}({\bar{\nu }},a)\, \bigg [{\bar{D}}_{{\bar{\nu }}}\bar{{\text{ tr }}}\,\sigma -3\, ({\bar{D}}_{{\bar{\nu }}}\sigma )({\bar{\nu }},{\bar{\nu }})\bigg ]\,\mathrm {d}{\bar{\mu }} \\ {}&\qquad \quad +4\,\int _{{\mathbb {R}}^3\setminus B_\lambda (0)} |x|^{-3}\, \bigg [{\bar{D}}_a\bar{{\text{ tr }}}\,\sigma -3\,|x|^{-2}\, ({\bar{D}}_a\sigma )(x,x)\bigg ] \,\mathrm {d}{\bar{\mu }} \\ {}&\qquad \quad +O(\lambda ^{-4}). \end{aligned} \end{aligned}$$

Since (*M*, *g*) is $C^4$-asymptotic to Schwarzschild, we obtain from (13) that

31 $$\begin{aligned} \sum _{i=1}^3 x^i\big [(\partial _i R)(x)+(\partial _i R)(-x)\big ]=O(|x|^{-5}). \end{aligned}$$

Indeed, if (31) failed, integration along radial lines would imply that (13) fails as well. Consequently, in conjunction with (14) and Lemma 12, we obtain

32 $$\begin{aligned} \int _{S_{{\tilde{\lambda }}}(\lambda \, \xi (\lambda ))}\,{\bar{g}}({\bar{\nu }},a)\,R\,\mathrm {d}{\bar{\mu }}= \int _{S_{\lambda }(\lambda \, \xi (\lambda ))}\,{\bar{g}}({\bar{\nu }},a)\,R\,\mathrm {d}{\bar{\mu }}+O(\lambda ^{-1}). \end{aligned}$$

Next, we observe that all derivatives of the form ${\bar{D}}^2_{{\bar{\nu }},{\bar{\nu }}}$ in the term

$$\begin{aligned}&\sum _{i=1}^3\bigg [ 2\,({\bar{D}}^2_{e_i,{\bar{\nu }}}\sigma )({\bar{\nu }},e_i)-({\bar{D}}^2_{e_i,e_i} \sigma )({\bar{\nu }},{\bar{\nu }})-({\bar{D}}^2_{{\bar{\nu }},{\bar{\nu }}}\sigma )(e_i,e_i)\\&\quad +\sum _{j=1}^3\big [({\bar{D}}^2_{e_i,e_i}\sigma )(e_j,e_j) -({\bar{D}}^2_{e_i,e_j}\sigma )(e_i,e_j)\big ]\bigg ] \end{aligned}$$

cancel. We may therefore use integration by parts and the decomposition $a=a^\perp +a^\top$ with respect to ${\bar{g}}$ to find that

33 $$\begin{aligned} \begin{aligned}&\int _{S_\lambda (0)}{\bar{g}}(\nu ,a)\,\sum _{i=1}^3\bigg [ 2\,({\bar{D}}^2_{e_i,{\bar{\nu }}}\sigma )({\bar{\nu }},e_i) -({\bar{D}}^2_{e_i,e_i}\sigma )({\bar{\nu }},{\bar{\nu }}) -({\bar{D}}^2_{{\bar{\nu }},{\bar{\nu }}}\sigma )(e_i,e_i)\\&\qquad +\sum _{j=1}^3\big [({\bar{D}}^2_{e_i,e_i}\sigma )(e_j,e_j)-({\bar{D}}^2_{e_i,e_j} \sigma )(e_i,e_j)\big ]\bigg ] \,\mathrm {d}{\bar{\mu }} \\&\quad =\lambda ^{-1}\int _{S_\lambda (0)}\bigg [{\bar{D}}_a\bar{{\text {tr}}}\, \sigma -({\bar{D}}_a\sigma )({\bar{\nu }},{\bar{\nu }})-2\,\lambda ^{-1}\,\bar{{\text {tr}}}\,\sigma \bigg ]\,\mathrm {d}{\bar{\mu }}. \end{aligned} \end{aligned}$$

Next, note that the vector field $Y=|x|^{-3}\,x$ is divergence free. Let

$$\begin{aligned} T=\sum _{i=1}^3\bigg [({\bar{D}}_Y\sigma )(a,e_i)+({\bar{D}}_a\sigma )(Y,e_i) -({\bar{D}}_{e_i}\sigma )(Y,a)-{\bar{g}}(Y,e_i)\,{\bar{D}}_a\bar{{\text {tr}}} \,\sigma \bigg ]e_i. \end{aligned}$$

There holds

$$\begin{aligned} \bar{{\text{ div }}}\,T=&|x|^{-3}\,\sum _{i=1}^3 \bigg [({\bar{D}}^2_{e_i,x}\sigma )(e_i,a)+({\bar{D}}^2_{e_i,a}\sigma ) (e_i,x)\\ {}&\qquad -({\bar{D}}^2_{e_i,e_i}\sigma )(x,a)-({\bar{D}}^2_{x,a}\sigma )(e_i,e_i)\bigg ] \\ {}&\quad +|x|^{-3}\,\big [{\bar{D}}_a\bar{{\text{ tr }}}\,\sigma -3\,|x|^{-2} \,({\bar{D}}_a\sigma )(x,x)\big ]. \end{aligned}$$

Consequently,

34 $$\begin{aligned} \begin{aligned}&\int _{{\mathbb {R}}^3\setminus B_\lambda (0)}|x|^{-3} \sum _{i=1}^3\bigg [({\bar{D}}^2_{e_i,x}\sigma )(e_i,a)+({\bar{D}}^2_{e_i,a} \sigma )(e_i,x)\\&\qquad -({\bar{D}}^2_{e_i,e_i}\sigma )(x,a)-({\bar{D}}^2_{x,a}\sigma ) (e_i,e_i)\bigg ]\,\mathrm {d}{\bar{\mu }} \\&\qquad +\int _{{\mathbb {R}}^3\setminus B_\lambda (0)} |x|^{-3}\, \bigg [{\bar{D}}_a\bar{{\text {tr}}}\,\sigma -3\,|x|^{-2}\, ({\bar{D}}_a\sigma )(x,x)\bigg ] \,\mathrm {d}{\bar{\mu }} \\&\quad =\,\lambda ^{-2}\int _{S_\lambda (0)} \bigg [{\bar{D}}_a\bar{{\text {tr}}} \,\sigma -({\bar{D}}_a\sigma )({\bar{\nu }},{\bar{\nu }})\bigg ]\,\mathrm {d}{\bar{\mu }}. \end{aligned} \end{aligned}$$

Finally, we use integration by parts and the decomposition $a=a^\perp +a^\top$ to find that

35 $$\begin{aligned} \begin{aligned}&2\, \lambda ^{-3}\int _{S_{\lambda }(0)}\bigg [\lambda \,({\bar{D}}_a\sigma ) ({\bar{\nu }},{\bar{\nu }})-6\,{\bar{g}}(a,{\bar{\nu }})\, \sigma ({\bar{\nu }},{\bar{\nu }})+3\,\sigma ({\bar{\nu }},a)\bigg ]\,\mathrm {d}{\bar{\mu }} \\ {}&\qquad +\,\lambda ^{-2}\int _{S_\lambda (0)} {\bar{g}}({\bar{\nu }},a)\, \bigg [{\bar{D}}_{{\bar{\nu }}}\bar{{\text{ tr }}}\,\sigma -3\, ({\bar{D}}_{{\bar{\nu }}}\sigma )({\bar{\nu }},{\bar{\nu }})\bigg ]\,\mathrm {d}{\bar{\mu }} \\ {}&\quad = \lambda ^{-2}\int _{S_\lambda (0)}\bigg [\bar{D}_a\bar{{\text{ tr }}}\, \sigma -({\bar{D}}_a\sigma )({\bar{\nu }},{\bar{\nu }})-2\,g(a,{\bar{\nu }})\, \bar{{\text{ tr }}}\,\sigma \bigg ]\,\mathrm {d}{\bar{\mu }} \\ {}&\qquad \quad +O(\lambda ^{-4}). \end{aligned} \end{aligned}$$

The assertion follows from assembling (28–35). $\square$

For the corollary below, recall the definition (2) of the Hamiltonian center $C=(C^1,\,C^2,\,C^3)$ of (*M*, *g*).

### Corollary 19

There holds, as $\lambda \rightarrow \infty$,

$$\begin{aligned} \lambda \,\xi (\lambda )=C+\frac{1}{128\,\pi }\,\lambda ^3\,\int _{S_{\lambda } (\lambda \,\xi (\lambda ))}R\,{\bar{\nu }}\,\mathrm {d}{\bar{\mu }}+o(1). \end{aligned}$$

### Proof

We define the quantities

$$\begin{aligned} \begin{aligned} z^\ell =\,&\frac{1}{32\,\pi }\,\lambda ^{-1}\,\int _{S_\lambda (0)} \bigg (\,\sum _{i,\,j=1}^3x^\ell \,x^j\,\bigg [(\partial _i\sigma )(e_i,e_j) -(\partial _j\sigma )(e_i,e_i)\bigg ] \\ {}&\qquad -\sum _{i=1}^3\bigg [x^i\,\sigma (e_i,e_\ell )-x^\ell \,\sigma (e_i,e_i)\bigg ]\bigg )\, \mathrm {d}{\bar{\mu }} \end{aligned} \end{aligned}$$

where $\ell =1,\,2,\,3$. Note that, by (2),

36 $$\begin{aligned} \lim _{\lambda \rightarrow \infty }z^\ell =C^\ell . \end{aligned}$$

Using integration by parts and the decomposition $e_\ell =e_\ell ^\perp +e_\ell ^\top$ with respect to ${\bar{g}}$, we obtain

37 $$\begin{aligned} z^\ell =\frac{1}{32\,\pi }\,\lambda \int _{S_\lambda (0)}\bigg [( \partial _{\ell }\sigma )({\bar{\nu }},\nu )- \partial _{\ell }\bar{{\text {tr}}}\,\sigma +2\,\lambda ^{-1} \,{\bar{\nu }}^\ell \,\bar{{\text {tr}}}\,\sigma \bigg ]\mathrm {d}\mu . \end{aligned}$$

The assertion follows from (36), (37), and Proposition 18. $\square$

### Proof of Theorem 2

Corollary 19 implies that

$$\begin{aligned} |\lambda \,\xi (\lambda )-C|\le \frac{1}{128\,\pi }\,\lambda ^3\,|\lambda \,\xi (\lambda )-C|^{-1} \int _{S_{\lambda }(\lambda \,\xi (\lambda ))}R\,{\bar{g}}(\lambda \, \xi (\lambda )-C,{\bar{\nu }})\,\mathrm {d}{\bar{\mu }}+o(1). \end{aligned}$$

Arguing as in the proof of Lemma 34 but using the stronger asymptotic conditions (8), we obtain

$$\begin{aligned} \frac{1}{128\,\pi }\,\lambda ^3\,|\lambda \,\xi (\lambda )-C|^{-1} \int _{S_{\lambda }(\lambda \,\xi (\lambda ))}R\,{\bar{g}}(\lambda \, \xi (\lambda )-C,{\bar{\nu }})\,\mathrm {d}{\bar{\mu }}\le o(1). \end{aligned}$$

Conversely, (13) and Lemma 12 imply that

38 $$\begin{aligned} |\Sigma _{\xi (\lambda ),\lambda }|^{-1}\int _{\Sigma _{\xi (\lambda ),\lambda }}x^\ell \, \mathrm {d}\mu =\lambda \,\xi (\lambda )+O(\lambda ^{-1}). \end{aligned}$$

The assertion follows from these estimates. $\square$

### Proof of Theorem 5

Let $\chi \in C^\infty ({\mathbb {R}})$ be such that $\chi (t)=1$ for all $t\in (3,5)$ and ${\text {supp}}(\chi )\subset [2,6]$. We define $\eta \in C^\infty ({\mathbb {R}}^3)$ by

$$\begin{aligned} \eta (x)=\sum _{k=0}^\infty \chi (10^{-k}\,|x|). \end{aligned}$$

Consider the metric

$$\begin{aligned} g=\left( 1+|x|^{-1}-\frac{1}{8}\,\eta (x)\,x^3\,|x|^{-4}\right) ^4{\bar{g}} \end{aligned}$$

on ${\mathbb {R}}^3\setminus \{0\}$. Note that

$$\begin{aligned} g=\bigg [(1+|x|^{-1})^{-4}+O(|x|^{-3})\bigg ]\,{\bar{g}}. \end{aligned}$$

It follows that the limit in (2) exists and that $C=0$.

Let $k\ge 1$ be an integer and suppose that $x\in {\mathbb {R}}^3$ with $3<10^{-k}\,|x|<5$. Using that $\eta =1$ near *x*, we compute

39 $$\begin{aligned} R(x)=\sum _{i=1}^3{\bar{D}}^2_{e_i,e_i}(x^3\,|x|^{-4})=4\,x^3\,|x|^{-6}. \end{aligned}$$

Conversely, if $6<10^{-k}\,|x|<8$, there holds $\eta = 0$ near *x*. We find that

$$\begin{aligned} R(x)=0. \end{aligned}$$

Let $\lambda _{k}=4\cdot 10^k$ and ${\hat{\lambda }}_{k}=7\cdot 10^k$. Using Lemma 12, that ${\bar{D}}R=O(|x|^{-6})$, and (39), we compute

$$\begin{aligned} \lambda _{k}^3\,\int _{S_{\lambda _{k},\xi (\lambda _{k})}}R\,{\bar{\nu }}\, \mathrm {d}{\bar{\mu }}=\lambda _{k}^3\,\int _{S_{\lambda _{k}}(0)}R\, {\bar{\nu }}\,\mathrm {d}{\bar{\mu }}+O(10^{-k}) = \frac{16\,\pi }{3}\,e_3+O(10^{-k}). \end{aligned}$$

In conjunction with $C=0$ and Corollary 19, we find

$$\begin{aligned} \lambda _k\,\xi (\lambda _k)=\frac{1}{24}\,e_3+O(10^{-k}). \end{aligned}$$

**Fig. 2:**
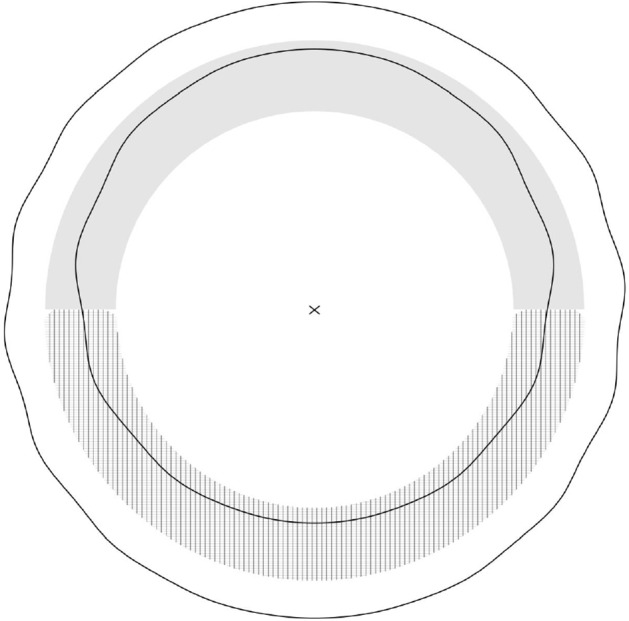
An illustration of the proof of Theorem 5. The scalar curvature is positive in the shaded region, negative in the hatched region, and vanishes elsewhere. The cross marks the Hamiltonian center of mass *C* in the asymptotically flat chart. The barycenter of the larger sphere $\Sigma _{{\hat{\lambda }}_k,\xi ({\hat{\lambda }}_k)}$ agrees with *C*. By contrast, the asymmetric distribution of scalar curvature moves the barycenter of the smaller sphere $\Sigma _{\lambda _k,\xi (\lambda _k)}$ away from *C*

Likewise, we obtain

$$\begin{aligned} {\hat{\lambda }}_k\,\xi ({\hat{\lambda }}_k)=O(10^{-k}); \end{aligned}$$

see Figure 2. It follows from this and (38) that the limit in (7) does not exist. $\square$

## Proof of Theorem 6

Throughout this section, we assume that (*M*, *g*) is $C^4$-asymptotic to Schwarzschild with mass $m=2$.

Let $\delta \in (0,1/2)$. We recall the definitions (69), (73), and (74) of the functions

$$\begin{aligned} G_\lambda ,\, G_1,\, G_{2,\lambda }:\{\xi \in {\mathbb {R}}^3:|\xi |<1-\delta \}\rightarrow {\mathbb {R}}. \end{aligned}$$

Moreover, recall from (72) that

$$\begin{aligned} G_\lambda (\xi )=G_1(\xi )+G_{2,\lambda }(\xi )+O(\lambda ^{-1}). \end{aligned}$$

According to Lemma 33, the function $G_1$ is strictly convex. We now identify a useful convexity criterion for functions that resemble $G_{2,\lambda }$.

### Lemma 20

Let $f\in C^{1}({\mathbb {R}}^3)$ be a non-negative function satisfying

40 $$\begin{aligned} \sum _{i=1}^3 x^i\,\partial _i(|x|^2\,f)\le 0. \end{aligned}$$

For every $\xi _1,\xi _2\in {\mathbb {R}}^3$ with $|\xi _1|,\,|\xi _2|<1$ and $\lambda >0$ there holds

$$\begin{aligned} \int _{S_{\xi _1,\lambda }}{\bar{g}}({\bar{\nu }},\xi _2-\xi _1)\,f\, \mathrm {d}{\bar{\mu }}\ge \int _{S_{\xi _2,\lambda }}{\bar{g}}({\bar{\nu }},\xi _2 -\xi _1)\,f\,\mathrm {d}{\bar{\mu }}. \end{aligned}$$

### Proof

By scaling, we may assume that $\lambda =1$. Moreover, we may assume that $\xi _2\ne \xi _1$. We define the hemispheres

$$\begin{aligned} S_+^\ell =\{x\in S_{1}(\xi _\ell ):{\bar{g}}({\bar{\nu }},\xi _2-\xi _1)\ge 0\} \quad \text {and} \quad S_-^\ell =\{x\in S_{1}(\xi _\ell ):{\bar{g}}({\bar{\nu }},\xi _2-\xi _1)\le 0\} \end{aligned}$$

where $\ell =1,2$. We choose an orthonormal basis $\{e_1,\,e_2,\,e_3\}$ of ${\mathbb {R}}^3$ with $e_1\perp {\text {span}}\{\xi _1,\xi _2\}$ and

$$\begin{aligned} e_3=\frac{\xi _2-\xi _1}{|\xi _2-\xi _1|} \end{aligned}$$

and parametrize almost all of $S_2^+$ via

$$\begin{aligned} \Psi :(0,\pi )\times (0,2\,\pi )\rightarrow S_2^+ \quad \text {given by}\quad \Psi (\zeta ,\varphi )= \xi _2+(\sin \zeta \,\sin \varphi ,\sin \zeta \,\cos \varphi ,\cos \zeta ). \end{aligned}$$

Likewise, we parametrize almost all of $S_1^+$ by

$$\begin{aligned} (0,\pi )\times (0,2\,\pi )\rightarrow S_1^+\qquad \text {where}\qquad (\theta ,\varphi )\mapsto \xi _1+(\sin \theta \,\sin \varphi ,\sin \theta \,\cos \varphi ,\cos \theta ). \end{aligned}$$

**Fig. 3:**
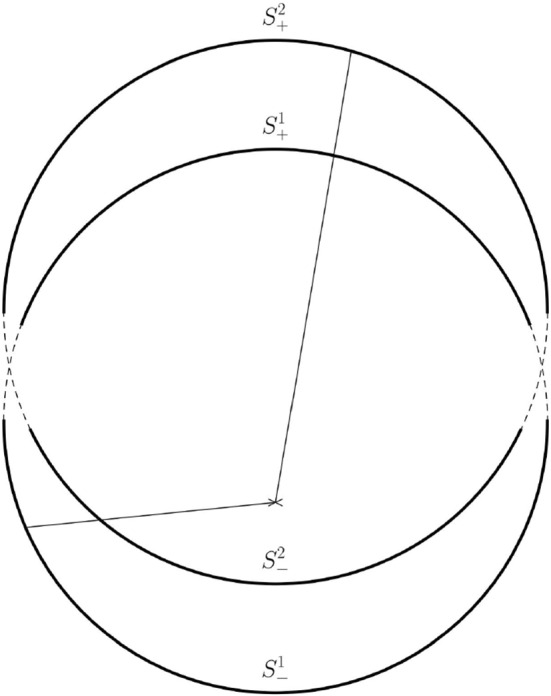
An illustration of the proof of Lemma 20. The function *f* is compared along the lines connecting $S^1_\pm$ and $S^2_\pm$. The cross marks the origin of ${\mathbb {R}}^3$

It is geometrically evident and straightforward to check that, given $\zeta$, there is $\theta =\theta (\zeta )$ with $\theta \le \zeta$ and $t=t(\zeta )>1$ such that

41 $$\begin{aligned} t\,\left[ \xi _1+(\sin \theta \,\sin \varphi ,\sin \theta \,\cos \varphi ,\cos \theta )\right] =\xi _2+(\sin \zeta \,\sin \varphi ,\sin \zeta ,\cos \varphi ,\cos \zeta ); \end{aligned}$$

see Figure 3. We define $a={\bar{g}}(\xi _1,e_3)$ and $b={\bar{g}}(\xi _2,e_3).$ Dotting (41) with $e_1$, we obtain

$$\begin{aligned} t=\frac{\sin \zeta }{\sin \theta }. \end{aligned}$$

Likewise, dotting (41) with $e_3$, we find that

$$\begin{aligned} t=\frac{\cos \zeta +b}{\cos \theta +a}. \end{aligned}$$

In particular, we obtain the relation

42 $$\begin{aligned} \frac{\cos \zeta +b}{\cos \theta +a}=\frac{\sin \zeta }{\sin \theta }. \end{aligned}$$

Differentiating (42) with respect to $\zeta$, we find

$$\begin{aligned} -\frac{\sin \zeta }{\cos \theta +a}+t\,\frac{\sin \theta }{\cos \theta +a}\,{\dot{\theta }} =\frac{\cos \zeta }{\sin \theta }-t\,\frac{\cos \theta }{\sin \theta }\,{\dot{\theta }}. \end{aligned}$$

Equivalently,

$$\begin{aligned} {\dot{\theta }} = t^{-1}\,\frac{\cos \theta \,\cos \zeta +\cos \zeta \,a+\sin \zeta \,\sin \theta }{1+a\,\cos \theta }. \end{aligned}$$

Using $\zeta \ge \theta$, we obtain that

$$\begin{aligned} \cos \theta \,\frac{\cos \theta \,\cos \zeta +\cos \zeta \,a+\sin \zeta \,\sin \theta }{1+a\,\cos \theta }\ge \cos \zeta . \end{aligned}$$

It follows that

$$\begin{aligned} {\dot{\theta }}\,\sin \theta \,\cos \theta \ge t^{-2}\, \sin \zeta \,\cos \zeta . \end{aligned}$$

Using that *f* is non-negative and (40), it follows that

43 $$\begin{aligned} \begin{aligned}&\int _{S^1_{+}}f\,{\bar{g}}({\bar{\nu }},\xi _2-\xi _1)\,\mathrm {d}{\bar{\mu }} -\int _{S^2_{+}}f\,{\bar{g}}({\bar{\nu }},\xi _2-\xi _1)\,\mathrm {d}{\bar{\mu }} \\&\quad \ge |\xi _2-\xi _1|\int _0^{2\,\pi }\int _0^\pi \left[ t^{-2}\,f(t^{-1}\,\Psi (\zeta ,\varphi ))-f(\Psi (\zeta ,\varphi )) \right] \,\sin \zeta \,\cos \zeta \,\mathrm {d}\zeta \,\mathrm {d}\varphi \\&\quad \ge 0. \end{aligned} \end{aligned}$$

The same argument shows that

44 $$\begin{aligned} \int _{S^1_{-}}f\,{\bar{g}}({\bar{\nu }},\xi _2-\xi _1)\,\mathrm {d}{\bar{\mu }} -\int _{S^2_{-}}f\,{\bar{g}}({\bar{\nu }},\xi _2-\xi _1)\,\mathrm {d}{\bar{\mu }}\ge 0. \end{aligned}$$

The assertion of the lemma follows from (43) and (44). $\square$

### Corollary 21

Let $\delta \in (0,1/2)$ and suppose that $f\in C^{1}({\mathbb {R}}^3)$ satisfies, as $x\rightarrow \infty$,

$$\begin{aligned} f\ge -o(|x|^{-4})\qquad \text {and}\qquad \sum _{i=1}^3x^i\,\partial _i(|x|^2\,f)\le o(|x|^{-2}). \end{aligned}$$

There holds, uniformly for all $\xi _1,\,\xi _2\in {\mathbb {R}}^3$ with $|\xi _1|,\,|\xi _2|<1-\delta$ as $\lambda \rightarrow \infty$,

$$\begin{aligned} \int _{S_{\xi _1,\lambda }}f\,{\bar{g}}({\bar{\nu }},\xi _2-\xi _1)\, \mathrm {d}{\bar{\mu }}\ge \int _{S_{\xi _2,\lambda }}f\,{\bar{g}}({\bar{\nu }},\xi _2-\xi _1)\, \mathrm {d}{\bar{\mu }}-o(\lambda ^{-2}). \end{aligned}$$

### Proof

This follows from Lemma 20 applied to the function $f_\epsilon =f+\epsilon \,|x|^{-4}$ for appropriate choice of $\epsilon >0$. $\square$

### Proof of Theorem 6

First, suppose that $R\ge - o(|x|^{-4})$. The argument presented in the proof of [12, Theorem 5] shows that there exists a family $\{\Sigma (\kappa ):\kappa \in (0,\kappa _0)\}$ of area-constrained Willmore spheres $\Sigma (\kappa )\subset M$ such that (4) holds with parameter $\kappa$ and such that (12) holds.

To prove the uniqueness statement, suppose that

$$\begin{aligned} \sum _{i=1}^3x^i\,\partial _i(|x|^2\,R)\le o(|x|^{-2}) \end{aligned}$$

and let $\delta \in (0,1/2)$. It follows from (72), Lemma 33, and Corollary 21 that $G_\lambda$ is strictly convex provided $\lambda >1$ is sufficiently large. In particular, $G_\lambda$ has at most one critical point. We can now argue exactly as in the proof of [12, Theorem 8]. $\square$

### Remark 22

Suppose that

$$\begin{aligned} \sum _{i=1}^3x^i\,\partial _i(|x|^2\,R)\le o(|x|^{-2}) \end{aligned}$$

and let $\xi (\lambda )\in {\mathbb {R}}^3$ be the unique critical point of $G_\lambda$ constructed in the proof of Theorem 6. Using Lemma 32, we find that

$$\begin{aligned} \xi (\lambda )=2\,\lambda ^2\, |({\bar{D}}G_1)({\xi (\lambda )})|^{-1}\,\int _{S_{\xi (\lambda ),\lambda }} R\,{\bar{\nu }}\,\mathrm {d}{\bar{\mu }}+O(\lambda ^{-1}). \end{aligned}$$

In particular, up to lower-order terms, the positioning of the asymptotic family by area-constrained Willmore surfaces (6) is determined by the asymptotic distribution of scalar curvature.

## Proof of Theorems 10 and 11

We recall the definitions (73) of $G_1$ and (74) of $G_{2,\lambda }$. A direct computation shows that

45 $$\begin{aligned} ({\bar{D}}G_1)({\xi })=2\,\pi \,\left[ 8\,(1-|\xi |)^{-2}+40\, (1-|\xi |)^{-1}-24\,\log (1-|\xi |)\right] \xi +O(1) \end{aligned}$$

as $$|\xi |\nearrow 1$$.

To prove Theorem 10 and Theorem 11, we construct suitable metrics *g* on ${\mathbb {R}}^3\setminus \{0\}$ such that the Schwarzschild contribution (45) cancels with that from $G_{2,\lambda }$ for suitable $\lambda >1$. We then adjust *g* accordingly to force the non-existence respectively existence of large area-constrained Willmore spheres.

First, we choose a function $\chi :{\mathbb {R}}\rightarrow [0,1]$ with ${\text {supp}}(\chi )\subset (1/2,4)$ and $\chi (t)=1$ if $t\in [3/4,3]$.

Let *k* and $\ell$ be non-negative integers. We define $\chi _k:{\mathbb {R}}\rightarrow [0,1]$ by

46 $$\begin{aligned} \chi _k(t)=\left\{ \!\begin{array}{ll} \chi (t) &{}\quad \text {if } t\le 1,\\ \\ 1 &{} \quad \text {if }1<t<k^2, \\ \\ \chi (k^{-2}\,t) &{}\quad \text {if } t>k^2. \end{array}\right. \end{aligned}$$

Note that

47 $$\begin{aligned} {\text {supp}}(\chi _k)\subset [1/2,4\,k^2]. \end{aligned}$$

Let $\lambda _{k,\ell }=k^2\,10^{\ell ^2}.$ Given $a_1,\,a_2,\,a_3,\,a_4\in {\mathbb {R}}$, we define

$$\begin{aligned} \eta _{k,\ell }=\,&\chi _k(10^{-\ell ^2}\,|x|)\bigg [a_1\,|x|^{-2}+a_2\,\lambda _{k,\ell }^{-1} \,|x|^{-1}\,\big (\log \lambda _{k,\ell }-\log |x|\big )\\\qquad&+a_3\,\lambda _{k,\ell }^{-2}\,\big (\log |x|-\log \lambda _{k,\ell }\big ) +a_4\,\lambda _{k,\ell }^{-5}\,(x^3)^3\bigg ]. \end{aligned}$$

Note that

48 $$\begin{aligned}&\lambda _{k,\ell }^{-1}\,|x|^{-1}\,|\log |x|-\log \lambda _{k,\ell }|< 100\,|x|^{-2} \quad \text {and}\nonumber \\&\lambda _{k,\ell }^{-2}\,|\log |x|-\log \lambda _{k,\ell }|< 100\,|x|^{-2} \end{aligned}$$

provided $1/2\,k^{-2}\le \lambda _{k,\ell }^{-1}\,|x|\le 4$. Using (46) and (48), we find that, for every multi-index *J*, there is a universal constant $c_J>1$ such that

49 $$\begin{aligned} |\partial _J\eta _{k,\ell }|\le c_J\,(|a_1|+|a_2|+|a_3|+|a_4|)\,|x|^{-2-|J|}. \end{aligned}$$

Let $x\in {\mathbb {R}}^3$ with $k^{-2}\,\le \lambda _{k,\ell }^{-1}\,|x|\le 2$. By (47), we have

50 $$\begin{aligned} \chi _k(10^{-j^2}\,|x|)=\delta _{\ell j} \end{aligned}$$

for every *j* provided $\ell$ is sufficiently large. Moreover, we compute

51 $$\begin{aligned} \sum _{i=1}^3({\bar{D}}^2_{e_i,e_i} \eta _{k,\ell })(x) =2\,a_1\,|x|^{-4}+a_2\,\lambda _{k,\ell }^{-1}\,|x|^{-3}+a_3\,\lambda _{k,\ell }^{-2} \,|x|^{-2}+6\,a_4\,\lambda _{k,\ell }^{-5}\,x^3. \end{aligned}$$

Fix $\xi \in {\mathbb {R}}^3$ with $|\xi |<1$. We compute

52 $$\begin{aligned} \begin{aligned}&\lambda _{k,\ell }^2\, \int _{S_{\xi ,\lambda _{k,\ell }}} \left[ 2\,a_1\,|x|^{-4}+a_2\,\lambda _{k,\ell }^{-1}\,|x|^{-3}+a_3\, \lambda _{k,\ell }^{-2}\,|x|^{-2}\right] {\bar{\nu }}\,\mathrm {d}{\bar{\mu }} \\&\quad = \int _{S_{1}(\xi )} \left[ 2\,a_1\,|x|^{-4}+a_2\,|x|^{-3}+a_3\,\,|x|^{-2}\right] {\bar{\nu }}\, \mathrm {d}{\bar{\mu }} \\&\quad = -2\,\pi \,\left[ a_1\,(1-|\xi |)^{-2}+(a_1+a_2)\,(1-|\xi |)^{-1}+(a_1-a_3)\,\log (1-|\xi |)\right] \xi \\&\qquad \quad +\sum _{i=1}^3a_i\,f_i(\xi )\,\xi \end{aligned} \end{aligned}$$

where $f_1,\,f_2,\,f_3\in C^\infty (B_1(0))$ are bounded. Likewise,

53 $$\begin{aligned} \lambda _{k,\ell }^2\,\int _{S_{\xi ,\lambda _{k,\ell }}}6\,a_4\,\lambda _{k,\ell }^{-5} \,x^3\,{\bar{\nu }}\,\mathrm {d}\mu =8\,\pi \,a_4\,e_3. \end{aligned}$$

Now, suppose that

$$\begin{aligned} g=\left( 1+|x|^{-1}+\frac{1}{2}\,\eta _{k,\ell }\right) ^4{\bar{g}}. \end{aligned}$$

Note that

$$\begin{aligned} R=-4\,\sum _{i=1}^3{\bar{D}}^2_{e_i,e_i}\eta _{k,\ell } \end{aligned}$$

and recall the definition (74) of $G_{2,\lambda _{k,\ell }}$. Assume that $|\xi |<1-k^{-2}$. Using (50), (51), (52), and (53), we conclude

54 $$\begin{aligned} \begin{aligned} ({\bar{D}}G_{2,\lambda _{k,\ell }})({\xi })=\,&-16\,\pi \,\bigg [a_1\,(1-|\xi |)^{-2}+(a_1+a_2)\,(1-|\xi |)^{-1}\\ {}&\qquad +(a_1-a_3)\,\log (1-|\xi |)\bigg ]\xi \\ {}&\quad +64\,\pi \,a_4\,e_3+8\,\sum _{i=1}^3a_i\,f_i(\xi )\,\xi \end{aligned} \end{aligned}$$

for every sufficiently large $\ell$. We emphasize the structural similarity of (45) and (54).

### Proof of Theorem 10

Let

$$\begin{aligned} g=\left( 1+|x|^{-1}+\frac{1}{2}\sum _{i=1}^\infty \eta _{i,i}\right) ^4\,{\bar{g}}. \end{aligned}$$

Using (49), we find that *g* is $C^k$-asymptotic to the Schwarzschild metric with mass $m=2$ for every $k\ge 2$.

We choose $a_1=1$, $a_2=4$ and $a_3=4$. Let $\delta \in (0,1/2)$. Recalling (72) and using (54) and (45), we obtain, uniformly for every $\xi \in {\mathbb {R}}^3$ with $|\xi |<1-\delta$ as $i\rightarrow \infty$,

55 $$\begin{aligned} ({\bar{D}} G_{\lambda _{i,i}})({\xi }) =64\,\pi \,a_4\,e_3+O(1). \end{aligned}$$

Suppose that there exists a family $\{\Sigma (\kappa ):\kappa \in (0,\kappa _0)\}$ of area-constrained Willmore spheres $\Sigma \subset {\mathbb {R}}^3\setminus \{0\}$ enclosing the origin and satisfying (4) with parameter $\kappa$ such that

$$\begin{aligned}&\lim _{\kappa \rightarrow 0} \rho (\Sigma (\kappa ))=\infty ,\qquad \limsup _{\kappa \rightarrow 0} \rho (\Sigma (\kappa ))^{-1}\,\lambda (\Sigma (\kappa ))<\delta ^{-1},\qquad \text {and}\\&\lim _{\kappa \rightarrow 0} \int _{\Sigma (\kappa )}|\mathring{h}|^2\,\mathrm {d}\mu =0. \end{aligned}$$

Arguing as in the proof of [12, Theorem 8], we find that the function $G_{\lambda _{i,i}}$ has a critical point $\xi _i$ with $|\xi _i|<1-\delta$ for every sufficiently large integer *i*. This is incompatible with (55) if $a_4>1$ is chosen sufficiently large. $\square$

For the proof of Theorem 11, we argue in two steps.

### Lemma 23

There are constants $c_J>1$ such that the following holds. For every $\delta \in (0,1/2)$, there exists a metric *g* on ${\mathbb {R}}^3\setminus \{0\}$ that is $C^k$-asymptotic to Schwarzschild with mass $m=2$ for every $k\ge 2$ with

56 $$\begin{aligned} \limsup _{x\rightarrow \infty }|x|^{2+|J|} \,|\partial _J \,\sigma |<c_J \end{aligned}$$

for every multi-index *J* that satisfies the following property.

There exists a sequence $\{\Sigma _i\}_{i=1}^\infty$ of area-constrained Willmore spheres $\Sigma _i\subset {\mathbb {R}}^3\setminus \{0\}$ such that

$$\begin{aligned} \lim _{i\rightarrow \infty } \rho (\Sigma _i)=\infty \end{aligned}$$

and $\lambda (\Sigma _i)^{-1}\,\Sigma _i$ converges smoothly to a round sphere while

$$\begin{aligned} \rho (\Sigma _i)<\delta \,\lambda (\Sigma _i) \qquad \text {and} \qquad m_H(\Sigma _i)>2 \end{aligned}$$

for all *i*.

### Proof

Let *k* be a positive integer and define the metric

$$\begin{aligned} g=\left( 1+|x|^{-1}+\frac{1}{2}\sum _{i=1}^\infty \eta _{k,i}\right) ^4{\bar{g}}. \end{aligned}$$

Note that (49) implies (56).

We choose $a_1=2$, $a_2=3$, $a_3=5$, and $a_4=0$. Using (45) and (54), we find that, uniformly for every $\xi \in {\mathbb {R}}^3$ with $|\xi |<1-k^{-2}$ as $k\rightarrow \infty$,

57 $$\begin{aligned} \sum _{j=1}^3\xi ^j\,(\partial _j [G_{1}+G_{2,\lambda _{k,i}}])({\xi }) =-16\,\pi \,(1-|\xi |)^{-2}+O(1) \end{aligned}$$

provided *i* is sufficiently large. Recalling (72), we conclude that for every large *k* there holds

58 $$\begin{aligned} \sum _{j=1}^3\xi ^j\,(\partial _j G_{\lambda _{k,i}})({\xi }) <0 \end{aligned}$$

for every sufficiently large *i* and every $\xi \in {\mathbb {R}}^3$ with $|\xi |=1-2\,k^{-2}$.

By contrast, it follows from (47) that $R(x)=0$ if $10^{-2\,i}<\lambda _{k,i}^{-1}\,|x|<1/2\,k^{-2}$. In conjunction with the estimate $R=O(|x|^{-4})$, we conclude from (74) that, as $i\rightarrow \infty$ for every $\xi \in {\mathbb {R}}^3$ with $1-1/2\,k^{-2}<|\xi |<1-10^{-2\,i}$,

$$\begin{aligned} ({\bar{D}} G_{2,\lambda _{k,i}})({\xi })=O(k^8). \end{aligned}$$

Recalling (45) and using (72), we conclude that there is $\delta (k)\in (0,k^{-2})$ such that

59 $$\begin{aligned} \sum _{j=1}^3\xi ^j\,(\partial _j G_{\lambda _{k,i}})(\xi )>0 \end{aligned}$$

for every $\xi \in {\mathbb {R}}^3$ with $|\xi |=1-\delta (k)$ and every sufficiently large integer *i*. Together with the fact that *g* is rotationally symmetric, (58) and (59) imply that for every *i* sufficiently large, $G_{\lambda _{k,i}}$ has a local minimum $\xi _i\in {\mathbb {R}}^3$ with $1-2\,k^{-2}<|\xi _i|<1$.

Finally, we observe that $G_{\lambda _{k,i}}(0)=O(1)$. Using (57) and (72), we conclude that $G_{\lambda _{k,i}}(\xi _i)<0$ for every sufficiently large *i*. The assertions of the lemma follow from Proposition 31, (69), and the definition of the Hawking mass (3). $\square$

### Proof of Theorem 11

Using Lemma 23, we may choose a sequence $\{g_{k}\}_{k=1}^\infty$ of Riemannian metrics $g_k$ on ${\mathbb {R}}^3\setminus \{ 0\}$ that are $C^k$-asymptotic to Schwarzschild with mass $m=2$ for every $k\ge 2$ and satisfy (56) such that the following holds. There is a sequence $\{\Sigma _k\}_{k=1}^\infty$ of spheres $\Sigma _k\subset {\mathbb {R}}^3\setminus \{0\}$ with the following four properties.

$$\circ $$
For every positive integer *k*, there holds
  60 $$\begin{aligned} \rho (\Sigma _{k+1})>10\,\Theta (\Sigma _k) \end{aligned}$$
  where $\Theta (\Sigma _k)=\sup \{|x|:x\in \Sigma _k\}$ is the outer radius of $\Sigma _k$.
$$\circ $$
$\Sigma _k$ is an area-constrained Willmore surface with Hawking mass $m_H(\Sigma _k)>2$ with respect to $g_k$.
$$\circ $$
$\lambda (\Sigma _k)^{-1}\,\Sigma _k$ converges smoothly to a round sphere.
$$\circ $$
There holds $\rho (\Sigma _k)<k^{-1}\, \lambda (\Sigma _k)$ for every positive integer *k*.

Now, we choose a smooth function $\gamma :{\mathbb {R}}\rightarrow [0,1]$ with ${\text {supp}}(\gamma )\subset [1/3,3]$ and $\gamma (t)=1$ for $t\in [1/2,2]$ and define $\gamma _k:{\mathbb {R}}\rightarrow [0,1]$

61 $$\begin{aligned} \gamma _k(t)=\left\{ \begin{array}{ll} \quad \gamma (\rho (\Sigma _k)^{-1}\,t) &{}\quad \text {if } t<\rho (\Sigma _k) \\ \\ \quad 1 &{}\quad \text {if } \rho (\Sigma _k)\le t \le \Theta (\Sigma _k)\\ \\ \quad \gamma (\Theta (\Sigma _k)^{-1}\,t)&{}\quad \text {if } t>\Theta (\Sigma _k). \end{array}\right. \end{aligned}$$

By (60), there holds ${\text {supp}}(\gamma _k)\cap {\text {supp}}(\gamma _j)=\emptyset$ whenever $k\ne j$. Consider the Riemannian metric

$$\begin{aligned} g=(1+|x|^{-1})^4\,{\bar{g}}+\sum _{k=0}^\infty \gamma _k(|x|)\,(g_k-(1+|x|^{-1})^4\,{\bar{g}}) \end{aligned}$$

on ${\mathbb {R}}^3\setminus \{0\}$. Using (56) and (61), we find that *g* is $C^k$-asymptotic to Schwarzschild with mass $m=2$ for every $k\ge 2$. Moreover, there holds $g=g_k$ near $\Sigma _k$. The assertions follow. $\square$

## The Hamiltonian Center of Mass

In this section, we recall some facts on the Hamiltonian center of mass of an asymptotically flat manifold (*M*, *g*).

Let (*M*, *g*) be a complete, non-compact Riemannian 3-manifold with integrable scalar curvature *R*. Given an integer $k\ge 2$ and $\tau >1/2$, we say that (*M*, *g*) is $C^k$-asymptotically flat of rate $\tau$ if there is a non-empty compact set whose complement in *M* is diffeomorphic to $\{x\in {\mathbb {R}}^3:|x|>1\}$ with, for every multi-index *J* with $|J|\le k$ and as $x\rightarrow \infty$,

$$\begin{aligned} g={\bar{g}}+\sigma \qquad \text {where}\qquad \partial _J\sigma =O(|x|^{-\tau -|J|}) \end{aligned}$$

in this asymptotically flat chart. We usually fix such an asymptotically flat chart and use it as reference for statements on the decay of quantities.

The mass *m* and the Hamiltonian center of mass $C=(C^1,\,C^2,\,C^3)$ of (*M*, *g*) are given by (1) and (2), respectively. The limit in (1) is well-defined for every such manifold (*M*, *g*). The limits in (2) exist if the metric *g* satisfies additional asymptotic symmetry conditions.

### Theorem 24

[16, Theorem 2.2]. Suppose that (*M*, *g*) is $C^2$-asymptotically flat of rate $\tau >1/2$ with, for every multi-index *J* with $|J|\le 2$ and as $x\rightarrow \infty$,

62 $$\begin{aligned} \partial _J\left[ g(x)-g(-x)\right]&=O(|x|^{-1-\tau -|J|}), \nonumber \\ R(x)-R(-x)&=O(|x|^{-7/2-\tau }). \end{aligned}$$

Then the Hamiltonian center of mass (2) of (*M*, *g*) is well-defined.

### Remark 25

If (*M*, *g*) is $C^2$-asymptotic to Schwarzschild, then (62) holds for every $\tau \in (1/2,1]$.

## The Geometric Center of Mass by Large Stable Constant Mean Curvature Spheres

The study of existence of large stable constant mean curvature spheres in asymptotically flat manifolds has been pioneered by Huisken and Yau [20]. There is a large body of important subsequent work; see e.g. [17, 23, 30] and the references therein. The following general existence result has been established by Nerz [24].

### Theorem 26

[24, Theorems 5.1 and 5.2]. Suppose that (*M*, *g*) is $C^2$-asymptotically flat of rate $\tau >1/2$ with positive mass $m>0$. Then there exists a number $H_0>0$ and a foliation of the complement of a compact subset of *M*

63 $$\begin{aligned} \{\Sigma _{CMC}(H):H\in (0,H_0)\} \end{aligned}$$

such that $\Sigma _{CMC}(H)$ is a stable constant mean curvature sphere with mean curvature *H* for every $H\in (0,H_0)$.

In [20, §4], Huisken and Yau have proposed to associate a geometric center of mass

$$\begin{aligned} C_{CMC}=(C_{CMC}^1,C_{CMC}^2,C_{CMC}^3) \end{aligned}$$

to the foliation (63) where

64 $$\begin{aligned} C_{CMC}^\ell =\lim _{H\searrow 0} |\Sigma _{CMC}(H)|^{-1}\int _{\Sigma _{CMC}(H)} x^\ell \,\mathrm {d}\mu , \end{aligned}$$

provided the limit on the right-hand side exists for $\ell =1,\,2,\,3$. The existence of these limits has been studied for instance by Metzger [23], Corvino and Wu [11], Huang [16], or Metzger and the first-named author [14]. In [24], Nerz has proven that the geometric center of mass (64) coincides with the Hamiltonian center of mass (2) of (*M*, *g*), provided *g* satisfies an additional asymptotic symmetry assumption.

### Theorem 27

[24, Theorem 6.3]. Suppose that, for every multi-index *J* with $|J|\le 2$ and as $x\rightarrow \infty$ ,

$$\begin{aligned} \partial _J\left[ g(x)-g(-x)\right]&=O(|x|^{-1/2-\tau -|J|}), \\ R(x)-R(-x)&=O(|x|^{-3- \tau }). \end{aligned}$$

Then the limits in (2) exist if and only if the limits in (64) exist in which case $C=C_{CMC}$.

### Remark 28

If (*M*, *g*) is $C^2$-asymptotic to Schwarzschild, then the assumptions of Theorem 27 are satisfied; see [16, Theorem 2].

### Remark 29

If (*M*, *g*) is $C^5$-asymptotic to Schwarzschild with non-negative scalar curvature *R* satisfying

$$\begin{aligned} \sum _{i=1}^3 x^i\,\partial _i(|x|^2\,R)\le 0, \end{aligned}$$

then the leaves of the foliation (63) are the only closed stable constant mean curvature surfaces $\Sigma \subset M$ with large enclosed volume; see [12] and also [6, 8, 9] for earlier work in this direction. According to Theorem 27 and Remark 28, the positioning of large stable constant mean curvature spheres is therefore governed by the Hamiltonian center of mass (2) of (*M*, *g*).

### Remark 30

In [13], the authors give short alternative proofs for Theorem 26 and Theorem 27 based on Lyapunov–Schmidt reduction. The analysis carried out there is much less technical than the one in Sect. 2.

## Lyapunov–Schmidt Reduction

We review the construction of the foliation by large area-constrained Willmore spheres (6) in [12]. Throughout, we will assume that (*M*, *g*) is $C^4$-asymptotic to Schwarzschild with scalar curvature *R*; see (5).

Let $\delta \in (0,1/2)$. Given $\lambda >1$ and $\xi \in {\mathbb {R}}^3$, we consider the spheres

65 $$\begin{aligned} S_{\xi ,\lambda }=\{x\in {\mathbb {R}}^3:|x-\lambda \,\xi |=\lambda \}. \end{aligned}$$

Given a function $u\in \Sigma _{\xi ,\lambda }$, let

66 $$\begin{aligned} \Sigma _{\xi ,\lambda }(u)=\{x+\lambda ^{-1}\,u(x)\,(x-\lambda \,\xi ):x\in S_{\xi ,\lambda }\} \end{aligned}$$

be the Euclidean graph of *u* over $S_{\xi ,\lambda }$. Moreover, let $\Lambda _1(S_{\xi ,\lambda })$ be the space of first spherical harmonics of $S_{\xi ,\lambda }$ and $\Lambda _1^\perp (S_{\xi ,\lambda })\subset C^\infty (S_{\xi ,\lambda })$ be its orthogonal complement with respect to $L^2(S_{\xi ,\lambda })$.

When stating that an error term

$$\begin{aligned} {\mathcal {E}}=O(\lambda ^{-l_1}\,|\xi |^{l_3})+O(\lambda ^{-l_2}) \end{aligned}$$

may be differentiated with respect to $\xi$ where $l_1,\,l_2>0$ and $l_3>1$, we mean that

$$\begin{aligned} \bar{D} {\mathcal {E}}= O(\lambda ^{-l_1}\,|\xi |^{l_3-1})+O(\lambda ^{-l_2}), \end{aligned}$$

where differentiation is with respect to $\xi$. When stating that ${\mathcal {E}}$ may be differentiated with respect to $\lambda$, we mean that

$$\begin{aligned} {\mathcal {E}}'= O(\lambda ^{-l_1-1}\,|\xi |^{l_3})+O(\lambda ^{-l_2-1}) \end{aligned}$$

Let $\Sigma \subset M$ be a closed surface with $\lambda (\Sigma )=\lambda$, i.e. $|\Sigma |=4\,\pi \,\lambda ^2$. The normalized Willmore energy of $\Sigma$ is given by

$$\begin{aligned} F_\lambda (\Sigma )=\lambda ^2\left( \int _{\Sigma }H^2\,\mathrm {d}\mu -16\,\pi -64\,\pi \, \lambda ^{-1}\right) . \end{aligned}$$

Note that area-constrained Willmore surfaces $\Sigma \subset M$ are area-constrained critical points of $F_\lambda$. Moreover, recall from e.g. [12, Appendix A] that such surfaces are either minimal or satisfy the constrained Willmore equation

$$\begin{aligned} \kappa (\Sigma )\,W(\Sigma )=H(\Sigma ) \end{aligned}$$

where

$$\begin{aligned} -W(\Sigma )=\Delta H+(|\mathring{h}|^2+{\text {Ric}}(\nu ,\nu ))\,H \end{aligned}$$

and

67 $$\begin{aligned} \kappa (\Sigma )=\left( \int _\Sigma H^2\,\mathrm {d}\mu \right) ^{-1}\int _{\Sigma } \left[ |\nabla H|^2-H^2\,|\mathring{h}|^2-H^2\,{\text {Ric}}(\nu ,\nu )\right] \mathrm {d}\mu . \end{aligned}$$

The Legendre polynomials $P_0,\,P_1,\,P_2,\dots$ are defined via a generating function. Given $s\in [0,1]$ and $t\in [0,1)$, there holds

$$\begin{aligned} (1-2\,s\,t+t^2)^{-\frac{1}{2}}=\sum _{\ell =0}^\infty P_l(s)\,t^\ell . \end{aligned}$$

The next proposition follows from [12, Proposition 17], [12, Lemma 20], and [12, Lemma 21].

### Proposition 31

There are constants $\lambda _0>1$, $c>1$, and $\epsilon >0$ depending only on (*M*, *g*) and $\delta \in (0,1/2)$ such that for every $\xi \in {\mathbb {R}}^3$ with $|\xi |<1-\delta$ and every $\lambda >\lambda _0$, there exists $u_{\xi ,\lambda }\in \Lambda _1^\perp (S_{\xi ,\lambda })$ and $\kappa _{\xi ,\lambda }\in {\mathbb {R}}$ such that the following hold. The surface

68 $$\begin{aligned} \Sigma _{\xi ,\lambda }=\Sigma _{\xi ,\lambda }(u_{\xi ,\lambda }) \end{aligned}$$

has area $|\Sigma _{\xi ,\lambda }|=4\,\pi \, \lambda ^2$. Moreover, $\Sigma _{\xi ,\lambda }$ is an area-constrained Willmore surface with parameter $\kappa _{\xi ,\lambda }$ if and only if $\xi$ is a critical point of the function

69 $$\begin{aligned} G_\lambda :\{\xi \in {\mathbb {R}}^3:|\xi |<1-\delta \}\rightarrow {\mathbb {R}}\qquad \text {given by} \qquad G_\lambda (\xi )=F_\lambda (\Sigma _{\xi ,\lambda }). \end{aligned}$$

There holds, uniformly for all $\xi \in {\mathbb {R}}^3$ with $|\xi |<1-\delta$ as $\lambda \rightarrow \infty$,

70 $$\begin{aligned} u_{\xi ,\lambda }&=-2+4\sum _{\ell =2}^\infty \frac{|\xi |^{\ell }}{\ell }\,P_l\left( -|\xi |^{-1}\,\bar{g}(y,\xi )\right) +O(\lambda ^{-1}), \end{aligned}$$

and

71 $$\begin{aligned} \kappa _{\xi ,\lambda }&=4\,\lambda ^{-3}+O(\lambda ^{-4}). \end{aligned}$$

The expansion (70) may be differentiated four times in spatial directions. The expansion (71) may be differentiated once with respect to $\lambda$.

If $\Sigma _{\xi ,\lambda }(u)$ with $u\in \Lambda ^\perp _1(S_{\xi ,\lambda })$ is an area-constrained Willmore surface with $|\Sigma _{\xi ,\lambda }(u)|=4\,\pi \, \lambda ^2$ and

$$\begin{aligned} |u|+\lambda \,|\nabla u|+\lambda ^2\,|\nabla ^2 u|+\lambda ^3\,|\nabla ^3 u|+\lambda ^4\,|\nabla ^4 u|&<\epsilon \,\lambda , \\ \lambda ^3\,|\kappa (\Sigma _{\xi ,\lambda }(u))|&<\epsilon \,\lambda , \end{aligned}$$

then $$u=u_{\xi ,\lambda }$$.

The leading order term of the function $G_\lambda$ can be computed explicitly.

### Lemma 32

[12, Lemma 22]. There holds, uniformly for every $\xi \in {\mathbb {R}}^3$ with $|\xi |<1-\delta$ as $\lambda \rightarrow \infty$,

72 $$\begin{aligned} G_\lambda (\xi )=G_1(\xi )+G_{2,\lambda }(\xi )+O(\lambda ^{-1}) \end{aligned}$$

where

73 $$\begin{aligned} G_1(\xi )=64\,\pi +\frac{32\,\pi }{1-|\xi |^2}-48\,\pi \,|\xi |^{-1}\log \frac{1+|\xi |}{1-|\xi |}-128\,\pi \log (1-|\xi |^2) \end{aligned}$$

and

74 $$\begin{aligned} G_{2,\lambda }(\xi )=2\,\lambda \,\int _{{\mathbb {R}}^3\setminus B_\lambda (\lambda \,\xi )}R\,\mathrm {d}{\bar{v}}. \end{aligned}$$

The expansion (72) may be differentiated twice with respect to $\xi$ and $\lambda$.

We record some properties of the function $G_\lambda$.

### Lemma 33

The function $G_1$ is strictly convex and strictly increasing in radial directions. Moreover, $G_1(0)=0$.

### Lemma 34

[12, Lemma 24]. Suppose that, as $x\rightarrow \infty$,

$$\begin{aligned} \sum _{i=1}^3x^i\,\partial _i(|x|^2\,R)\le o(|x|^{-2})\qquad \text {and}\qquad R(x)-R(-x)=o(|x|^{-4}). \end{aligned}$$

Then, uniformly for every $\xi \in {\mathbb {R}}^3$ with $|\xi |<1-\delta$ as $\lambda \rightarrow \infty$,

$$\begin{aligned}|\xi |^{-1}\,\sum _{i=1}^3\xi ^i\,(\partial _iG_{2,\lambda })({\xi })\ge -o(1).\end{aligned}$$

If the stronger decay assumptions

$$\begin{aligned} \sum _{i=1}^3x^i\,\partial _i(|x|^2\,R)\le O(|x|^{-3})\qquad \text {and}\qquad R(x)-R(-x)=O(|x|^{-5}) \end{aligned}$$

hold, then, uniformly for every $\xi \in {\mathbb {R}}^3$ with $|\xi |<1-\delta$ as $\lambda \rightarrow \infty$,

$$\begin{aligned} |\xi |^{-1}\,\sum _{i=1}^3\xi ^i\,(\partial _iG_{2,\lambda })({\xi })\ge -O(\lambda ^{-1}). \end{aligned}$$

### Proof

This follows exactly as in [12, Lemma 24]. $\square$

The following proposition is a consequence of Lemma 32, Lemma 33, and Lemma 34.

### Proposition 35

[12, Theorems 5 and 8]. Suppose that, as $x\rightarrow \infty$,

$$\begin{aligned} \sum _{i=1}^3 x^i\,\partial _i(|x|^2\,R)\le o(|x|^{-2})\qquad \text {and}\qquad R(x)-R(-x)=o(|x|^{-4}). \end{aligned}$$

There is $\lambda _0>1$ such that for every $\lambda >\lambda _0$ the function $G_\lambda$ has a unique critical point $\xi (\lambda )$. Moreover, as $\lambda \rightarrow \infty$,

$$\begin{aligned} \xi (\lambda )=o(1). \end{aligned}$$

### Remark 36

It follows from (71) that the function

$$\begin{aligned} \lambda \mapsto \kappa (\Sigma (\lambda )) \end{aligned}$$

is decreasing on $(\lambda _0,\infty )$, provided $\lambda _0>1$ is sufficiently large.

## Some Geometric Expansions

We collect several geometric computations that are needed in this paper. Throughout this section, we assume that (*M*, *g*) is $C^4$-asymptotic to Schwarzschild with mass $m=2$. We use a bar to indicate that a geometric quantity has been computed with respect to the Euclidean background metric ${\bar{g}}$. Likewise, we use the subscript *S* to indicate that the Schwarzschild metric

$$\begin{aligned} g_S=(1+|x|^{-1})^4\,{\bar{g}} \end{aligned}$$

with mass $m=2$ has been used in the computation.

Let $\delta \in (0,1/2)$, $\xi \in {\mathbb {R}}^3$ with $|\xi |<1-\delta$, and $\lambda >\lambda _0$, where $\lambda _0$ is the constant from Proposition 31. Recall that $S_{\xi ,\lambda }=S_\lambda (\lambda \,\xi )$.

The estimates below depend on $\delta \in (0,1/2)$ and $\lambda _0>1$ but are otherwise independent of $\lambda$ and $\xi$.

### Lemma 37

There holds

75 $$\begin{aligned} {\text {Ric}}_S(e_i,e_j)=2\,|x|^{-3}\,(\delta _{ij}-3\,x^i\,x^j\,|x|^{-2}) +O(|x|^{-4}) \end{aligned}$$

and, as $$x\rightarrow \infty $$,

76 $$\begin{aligned} \begin{aligned}&({\text {Ric}}-{{\text {Ric}}_S})(e_i,e_j)\\&\quad =\,\frac{1}{2} \,\sum _{k=1}^3\left[ ({\bar{D}}^2_{e_k,e_i}\sigma )(e_k,e_j)+({\bar{D}}^2_{e_k,e_j}\sigma )(e_k,e_i)\right. \\&\left. \qquad \qquad -({\bar{D}}^2_{e_k,e_k} \sigma )(e_i,e_j)-({\bar{D}}^2_{e_i,e_j}\sigma )(e_k,e_k)\right] +O(|x|^{-5}). \end{aligned} \end{aligned}$$

### Proof

(75) follows from a direct computation. To prove (76), we define the family of metrics $g_t=g_S+t\,\sigma ,$ where $t\in [0,1]$. Note that $g_0=g_S$ and $g_1=g$. The estimate follows upon linearization of the expression

$$\begin{aligned} {\text {Ric}}(e_i,e_j)=\sum _{k=1}^3\bigg [\partial _k \Gamma ^k_{ij}-\partial _i\Gamma ^k_{kj}+\sum _{\ell =1}^3\big [\Gamma ^k_{k\ell }\, \Gamma ^\ell _{ij}-\Gamma ^{k}_{il}\,\Gamma ^\ell _{kj}\big ]\bigg ] \end{aligned}$$

where $\Gamma ^{k}_{ij}$ are the Christoffel symbols of *g*. $\square$

### Lemma 38

[12, Lemma 41]. There holds

$$\begin{aligned} \nu _S(S_{\xi ,\lambda })=(1+|x|^{-1})^{-2}\,{\bar{\nu }} \end{aligned}$$

and, uniformly for every $\xi \in {\mathbb {R}}^3$ with $|\xi |<1-\delta$ as $\lambda \rightarrow \infty$,

77 $$\begin{aligned} \nu -\nu _S=\,\frac{1}{2}\,\sigma ({\bar{\nu }},{\bar{\nu }})\,{\bar{\nu }} -\sum _{i=1}^3\sigma ({\bar{\nu }},e_i)\,e_i+O(\lambda ^{-3}). \end{aligned}$$

Given $\xi \in {\mathbb {R}}^3$ and $\lambda >1$, we define the vector field

78 $$\begin{aligned} Z_{\xi ,\lambda }=(1+|x|^{-1})^{-2}\,\lambda ^{-1}\,(x-\lambda \,\xi ). \end{aligned}$$

Note that $Z_{\xi ,\lambda }=\nu _S(S_{\xi ,\lambda })$ on $S_{\xi ,\lambda }$.

For the statement of the next lemma, recall the definition of the conformal Killing operator ${\mathcal {D}}$ given by

79 $$\begin{aligned} ({\mathcal {D}}Z)(X,Y)=g(D_XZ,Y)+g(D_YZ,X)-\frac{2}{3}\,{\text {div}}(Z)\,g(X,Y) \end{aligned}$$

for vector fields $Z,\,X,\,Y$.

### Lemma 39

There holds

80 $$\begin{aligned} \begin{aligned} ({\mathcal {D}}_S Z_{\xi ,\lambda })(e_i,e_j)=\,&4\,\lambda ^{-1}\,|x|^{-3}\,x^i\,x^j-2\,|x|^{-3} \,\left( x^i\,\xi ^j+\xi ^i\,x^j\right) \\ {}&\quad +\frac{4}{3}\,\left( |x|^{-3}\, {\bar{g}}(x,\xi )-\lambda ^{-1}\,|x|^{-1}\right) \,\delta _{ij}\\ {}&\quad +O(|x|^{-3})+O(\lambda ^{-1}\,|x|^{-2}) \end{aligned} \end{aligned}$$

and, uniformly for every $\xi \in {\mathbb {R}}^3$ with $|\xi |<1-\delta$ as $\lambda \rightarrow \infty$ on ${\mathbb {R}}^3\setminus B_{\lambda }(\lambda \,\xi )$,

81 $$\begin{aligned} \begin{aligned}&({\mathcal {D}}Z_{\xi ,\lambda }-{\mathcal {D}}_SZ_{\xi ,\lambda })(e_i,e_j)\\ {}&\quad =\,\sum _{k=1}^3\bigg [(\lambda ^{-1}\,x^k-\xi ^k)\,(\partial _k\sigma )(e_i,e_j) -\frac{1}{3}\,(\lambda ^{-1}\,x^k-\xi ^k)\,(\partial _k\bar{{\text{ tr }}}\, \sigma )\,\delta _{ij}\bigg ]\\ {}&\qquad \quad +O(\lambda ^{-1}\,|x|^{-3})+O(|x|^{-4}). \end{aligned} \end{aligned}$$

### Proof

To prove (80), we expand, as $\lambda \rightarrow \infty$ on ${\mathbb {R}}^3\setminus B_{\lambda }(\lambda \,\xi )$,

$$\begin{aligned} Z_{\xi ,\lambda }=\lambda ^{-1}\,x-\xi -2\,\lambda ^{-1}\,|x|^{-1}\,x+2\,|x|^{-1} \,\xi +O(\lambda ^{-1}\,|x|^{-1})+O(|x|^{-2}). \end{aligned}$$

The first two terms on the right-hand side are conformal Killing vector fields. For the third term, we compute

$$\begin{aligned} ({\mathcal {D}}_S(\lambda ^{-1}\,|x|^{-1}\,x))(e_i,e_j)=\,&\lambda ^{-1}\,(\bar{{\mathcal {D}}}(|x|^{-1}\,x))(e_i,e_j)+O(\lambda ^{-1}\,|x|^{-2}) \\ =\,&\frac{2}{3}\,\lambda ^{-1}\,|x|^{-1}\,\delta _{ij}-2\,\lambda ^{-1}\,|x|^{-3}\,x^i\,x^j. \end{aligned}$$

Likewise, for the fourth term, we compute

$$\begin{aligned} ({\mathcal {D}}_S(|x|^{-1}\,\xi ))(e_i,e_j)=\,&(\bar{{\mathcal {D}}}(|x|^{-1}\, \xi ))(e_i,e_j)+O(|x|^{-3})\\ =\,&-|x|^{-3}\,\big [{\bar{g}}(x,e_i)\,{\bar{g}}(\xi ,e_j)+{\bar{g}}(\xi ,e_i) \,{\bar{g}}(x,e_j)\big ]\\ {}&\quad +\frac{2}{3}\,|x|^{-3}\,{\bar{g}}(x,\xi )\,\delta _{ij}+O(|x|^{-3}). \end{aligned}$$

To prove (81), we again consider the family of metrics $g_t=g_S+t\,\sigma ,$ where $t\in [0,1]$, and linearize the expression

$$\begin{aligned} ({\mathcal {D}}Z_{\xi ,\lambda })(e_i,e_j)=g(D_{e_i}Z_{\xi ,\lambda },e_j) +g(D_{e_j}Z_{\xi ,\lambda },e_i)-\frac{2}{3}\,g(e_i,e_j)\, {\text {div}}Z_{\xi ,\lambda }. \end{aligned}$$

$$\square $$

We recall the following expansion of the Willmore operator of a sphere $S_{\xi ,\lambda }$.

### Lemma 40

[12, Corollary 45]. There holds, uniformly for every $\xi \in {\mathbb {R}}^3$ with $|\xi |<1-\delta$ as $\lambda \rightarrow \infty$,

$$\begin{aligned} {W}({{S}_{\xi ,\lambda }})=4\,\lambda ^{-4}\sum _{\ell =0}^\infty (\ell -1)\,(\ell +1)\,(\ell +2)\,|\xi |^{\ell }\,P_l(-|\xi |^{-1}\,{\bar{g}} ({\bar{\nu }},\xi ))+{O}(\lambda ^{-5}). \end{aligned}$$

The following estimate is contained in the proof of [12, Lemma 42].

### Lemma 41

There holds, uniformly for every $\xi \in {\mathbb {R}}^3$ with $|\xi |<1-\delta$ as $\lambda \rightarrow \infty$,

$$\begin{aligned} \int _{S_{\xi ,\lambda }} |\mathring{h}|^2\,\mathrm {d}\mu =O(\lambda ^{-4}). \end{aligned}$$

This identity may be differentiated once with respect to $\xi$.

The next lemma follows from Taylor’s theorem and [12, Lemma 31].

### Lemma 42

There exists a constant $\lambda _0>1$ such that for every $\lambda >\lambda _0$ the following holds. Let $u\in C^\infty (S_{\xi ,\lambda })$ and suppose that there is $\epsilon >0$ with

$$\begin{aligned} | u|+\lambda \,|\nabla u|+\lambda ^2\,|\nabla ^2 u|+\lambda ^3\,|\nabla ^3 u|+\lambda ^4\, |\nabla ^4 u|\le \epsilon . \end{aligned}$$

Then we have, uniformly for every $\xi \in {\mathbb {R}}^3$ with $|\xi |<1-\delta$ as $\lambda \rightarrow \infty$,

$$\begin{aligned} \int _{\Sigma _{\xi ,\lambda }(u)}H^2\,\mathrm {d}\mu -\int _{S_{\xi ,\lambda }}H^2\, \mathrm {d}\mu =-2\,\int _{S_{\xi ,\lambda }}W\,u\,\mathrm {d}\mu +O(\epsilon ^2\,\lambda ^{-2}). \end{aligned}$$

This identity may be differentiated once with respect to $\xi$.
